# Strong acceptor incorporated phenothiazine-C_60_ multi-redox push–pull conjugates: demonstration of C_60_'s superior electron acceptor characteristics†

**DOI:** 10.1039/d5sc02950c

**Published:** 2025-05-29

**Authors:** Pankaj K. Gupta, Chamari V. Ileperuma, Rajneesh Misra, Francis D'Souza

**Affiliations:** a Department of Chemistry, Indian Institute of Technology Indore Indore 453552 India rajneeshmisra@iiti.ac.in; b Department of Chemistry, University of North Texas at Denton 1155 Union Circle, #305070 Denton TX 76203-5017 USA Francis.DSouza@unt.edu

## Abstract

Among the several exceptional properties of fullerene, C_60_, its electron acceptor property is a highly studied topic. This work demonstrates the superior electron acceptor property of C_60_, even in the presence of a stronger electron acceptor(s) in multi-modular donor–acceptor constructs. For this, novel bis-phenothiazine-C_60_ donor–acceptor conjugates incorporating strong electron acceptors, tetracyanobutadiene (TCBD) or dicyanoquinodimethane (DCNQ), have been newly synthesized. In this molecular design, the TCBD and DCNQ electron acceptors were placed between the two phenothiazine entities, while the C_60_ was in the peripheral position of one of the phenothiazine entities. After establishing their molecular structure, intramolecular charge transfer in these systems was probed through optical and electrochemical measurements, while time-dependent DFT studies initially probed the ground and excited-state charge transfer. These studies established the role of C_60_ as an acceptor compared to TCBD and DCNQ due to the sandwiching of the latter electron acceptors between two phenothiazine electron donors, which modulates their overall electron-acceptor abilities. Femtosecond pump–probe studies, covering broad spatial and temporal scales, provided experimental evidence that C_60_ serves as the terminal electron acceptor, wherein the electron transfer product of C_60_ was spectrally possible to identify. These unprecedented findings present new opportunities for designing multi-redox entities featuring push–pull systems, paving the way for the next generation of efficient energy harvesting, photocatalytic, and optoelectronic applications.

## Abstract 2

Novel bis-phenothiazine-C_60_ conjugates, incorporating strong electron acceptors, TCBD or DCNQ, between the two phenothiazine entities, have been newly synthesized. The involvement of C_60_ as the terminal electron acceptor is demonstrated.

## Graphical abstract

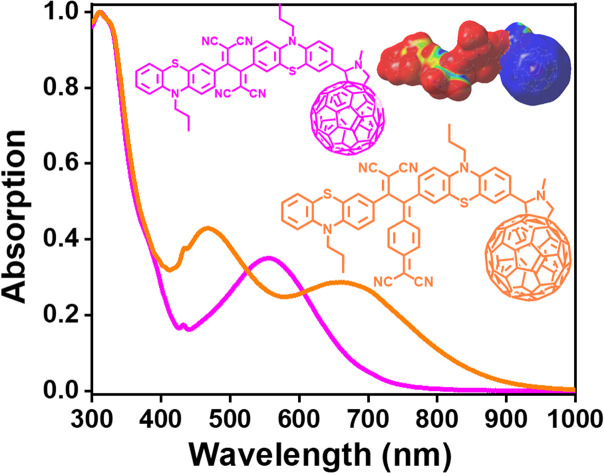

## Introduction

In donor–acceptor conjugates exhibiting excited charge transfer (CT),^1,2^ fullerene, C_60_, has significantly impacted the research area due to its facile reduction and low-energy demand in electron transfer reactions.^3–5^ Consequently, many covalently and non-covalently linked systems have been constructed and studied to understand the fundamental mechanistic details of electron transfer and the subsequent building of energy harvesting devices.^6–11^ Another class of strong electron acceptors that have recently gained momentum are nonplanar push–pull substituted but-1,3-dienes which were obtained by [2 + 2] cycloaddition retroelectrocyclization reaction of either tetracyanoethene (TCNE) or 7,7,8,8-tetracyano-*p*-quinodimethane (TCNQ) with electron–donor (D) substituted alkynes, resulting in tetracyanobutadiene (TCBD) and dicyanodiquinodimethane (DCNQ) incorporated push–pull sys-tems.^12–23^ These reactions proceed almost quantitatively when alkynyl functionalized electron donors are used, and consequently, they have been recognized as a metal-free click chemistry. Strong intramolecular charge transfer (ICT) has been reported for this class of compounds due to their strong push–pull characteristics.^12–23^

**Fig. 1:**
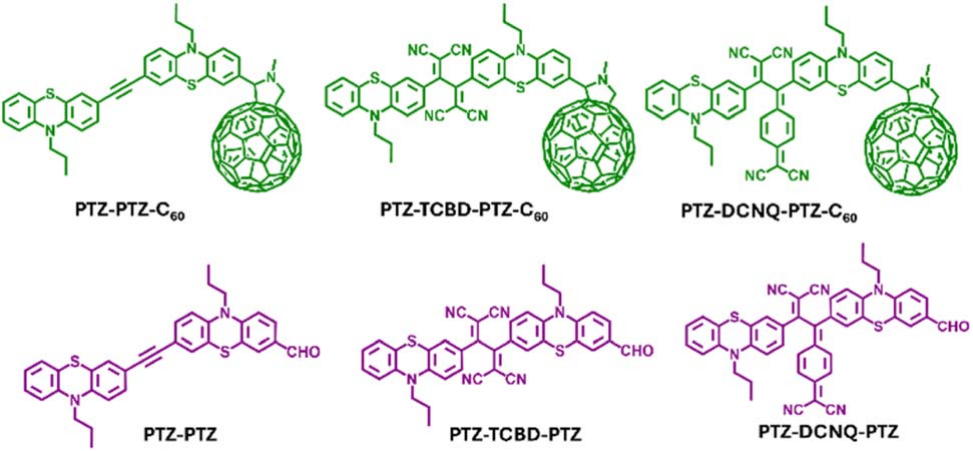
Structures and abbreviations of the investigated compounds along with the controls in the present study.

Combining multiple donors and multiple acceptors often results in new ground and excited state events which could provide new research directions and transform the entire research field;^17–19^ however, designing and synthesizing such multi-modular systems is challenging, requiring several reaction steps, which hampers research progress. With our quest to build multi-modular push–pull systems revealing new redox and photochemical phenomena, we present a study in which we constructed multi-modular conjugates, whose structures are shown in Fig. 1. In our design, two entities of a well-known electron donor, phenothiazine,^17^ are used to house the TCBD/DCNQ electron acceptor at the center and C_60_ at one of the phenothiazines. As demonstrated here, the first reduction of C_60_ is harder than that of TCBD and DCNQ, making an interesting class of nanocarbon-carrying push–pull systems. As a result of these unique structures, tuning of the electronic structure results in substantial changes in the ground (charge polarization) and excited state charge transfer are witnessed, revealing their role in controlling push–pull charge transfer events. Key findings on this fascinating class of molecular systems are summarized below.

## Results and discussion

### Synthesis

**Scheme 1:**
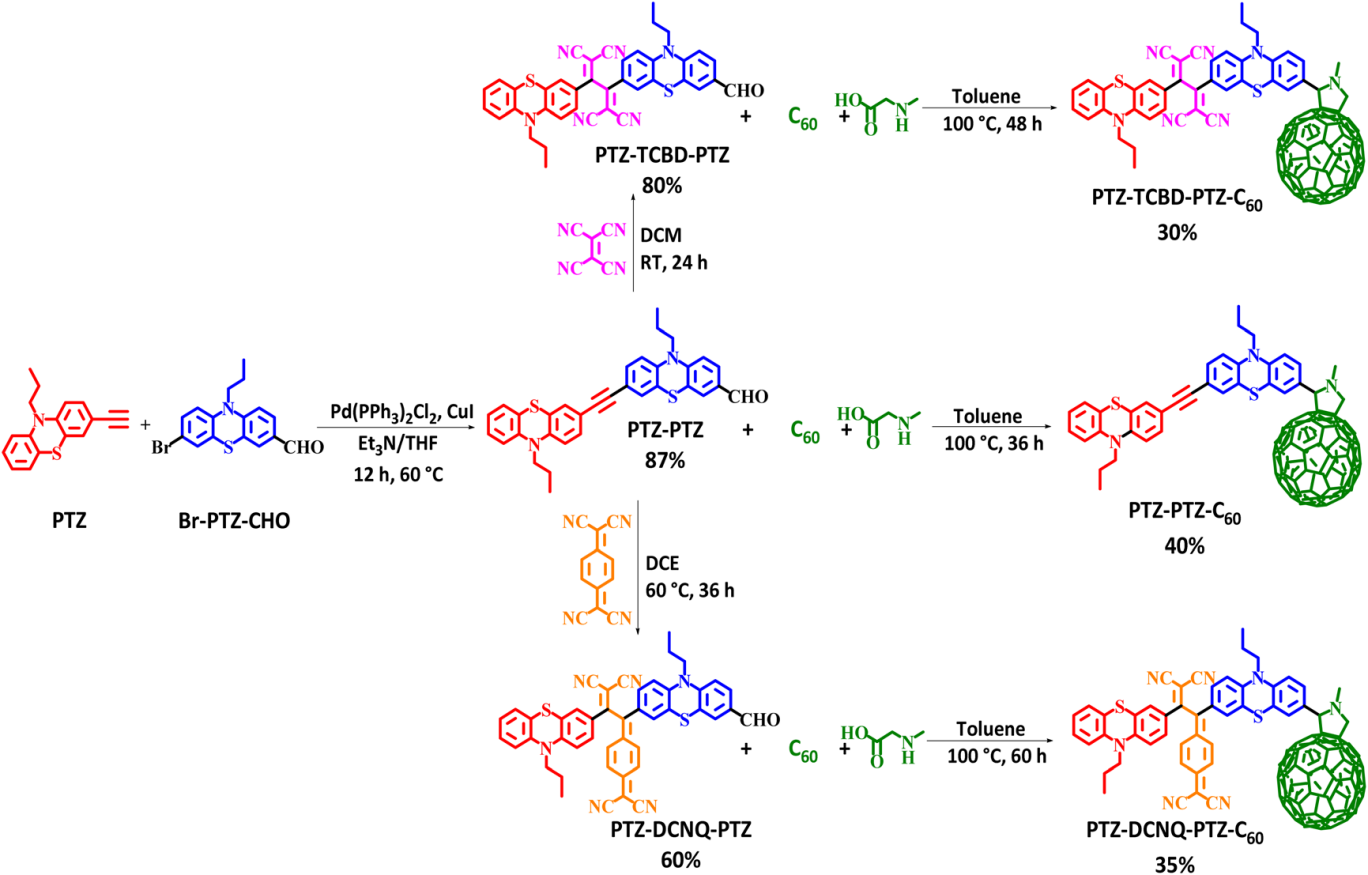
Synthetic route of phenothiazine chromophores PTZ-PTZ, PTZ-TCBD-PTZ and PTZ-DCNQ-PTZ and their C_60_ substituted derivatives PTZ-PTZ-C_60_, PTZ-TCBD-PTZ-C_60_ and PTZ-DCNQ-PTZ-C_60_.

The donor–acceptor based phenothiazine chromophores PTZ–PTZ, PTZ-TCBD-PTZ and PTZ-DCNQ-PTZ were synthesized by the Sonogashira cross-coupling followed by [2 + 2] cycloaddition–retroelectrocyclization reaction using donor phenothiazine (PTZ), and acceptors tetracyanoethylene (TCNE) or 7,7,8,8-tetracyanoquino-dimethane (TCNQ) units. The C_60_ derivatives PTZ-PTZ-C_60_, PTZ-TCBD-PTZ-C_60_ and PTZ-DCNQ-PTZ-C_60_ were synthesized by 1,3-dipolar cycloaddition reaction using a fullerene acceptor.^17–19^ The details are outlined in Scheme 1. The reaction of 3-ethynyl-10-propyl-10*H*-phenothiazine PTZ with 1.1 equivalent of 7-bromo-10-propyl-10*H*-phenothiazine-3-carbaldehyde Br-PTZ-CHO using THF : TEA (1 : 1) under N_2_ atmosphere in the presence of Pd(PPh_3_)_2_Cl_2_/CuI *via* Sonogashira cross-coupling reaction, resulted phenothiazine chromophore PTZ-PTZ in 85% yield. The TCBD and DCNQ incorporated phenothiazine chromophores PTZ-TCBD-PTZ and PTZ-DCNQ-PTZ were designed and synthesized by the [2 + 2] cycloaddition–retroelectrocyclization reaction. Compound PTZ-PTZ was reacted with 1.1 equivalents of TCNE in dichloromethane at room temperature for 24 h to produce PTZ-TCBD-PTZ in 80% yield. Similarly, PTZ-PTZ was reacted with 1.1 equivalents of TCNQ in dichloroethane (DCE) for 36 h at 60 °C, resulting PTZ-DCNQ-PTZ in 60% yield. Subsequently, chromophores PTZ-PTZ, PTZ-TCBD-PTZ and PTZ-DCNQ-PTZ were reacted with fullerene (C_60_) in toluene in the presence of an excess amount of *N*-methyl glycine (sarcosine) followed by a 1,3-dipolar cycloaddition reaction (Prato reaction)^24^ resulting in fulleropyrrolidine derivatives PTZ-PTZ-C_60_, PTZ-TCBD-PTZ-C_60_ and PTZ-DCNQ-PTZ-C_60_ in 40%, 30%, and 35% yields, respectively. The details of synthesis and characterization are summarized in the (ESI along with ^1^H, ^13^C, and mass spectra; see Fig. S1–S18 in ESI).†

### Spectroscopic studies

**Fig. 2:**
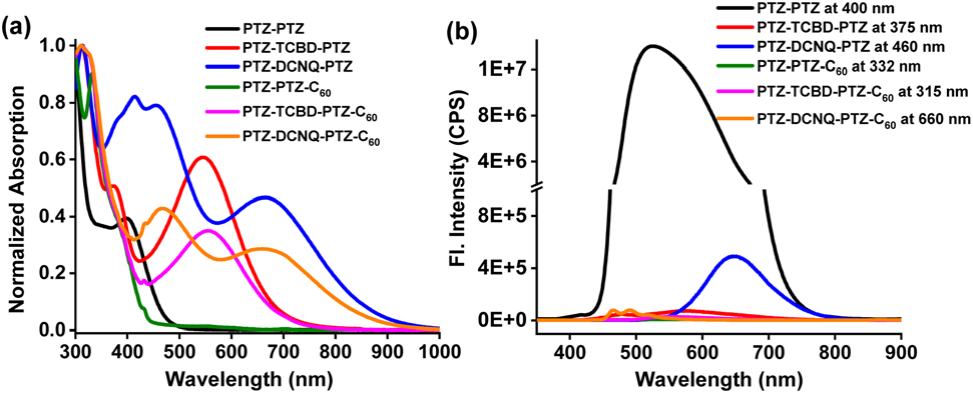
(a) Absorption and (b) fluorescence spectrum of the indicated compounds in dichlorobenzene (excitation wavelengths are also indicated).

Fig. 2a shows the absorption spectra of the compounds investigated in *o*-dichlorobenzene (DCB). No CT peaks for PTZ-PTZ and PTZ-PTZ-C_60_ lacking TCBD or DCNQ in the visible region were observed. For these, the main peaks appeared in the 300–400 nm range due to π–π* transitions of PTZ and C_60_ (for PTZ-PTZ-C_60_). For PTZ-TCBD-PTZ and PTZ-DCNQ-PTZ, having strong electron acceptors TCBD and DCNQ in proximity, a strong CT peak with peak maxima at 548 nm for PTZ-TCBD-PTZ and 667 nm for PTZ-DCNQ-PTZ was observed, signifying the importance of these electron acceptors in ground state charge polarization, *i.e.*, the existence of PTZ^*δ*+^-TCBD^*δ*−^-PTZ^*δ*+^ and PTZ^*δ*+^-DCNQ^*δ*−^-PTZ^*δ*+^ quadrupolar states. Upon covalent linkage of C_60_, the CT band in PTZ-TCBD-PTZ-C_60_ and PTZ-DCNQ-PTZ-C_60_, having all three PTZ, TCBD/DCNQ, and C_60_ entities, revealed a substantial reduction in CT peak intensity (about 40%), indicating CT modulation involving C_60_, likely by forming PTZ^*δ*+^-TCBD^*δ*−^-PTZ^*δ*+^-C_60_^*δ*−^ and PTZ^*δ*+^-DCNQ^*δ*-^-PTZ^*δ*+^-C_60_^*δ*−^ type bis-bipolar CT states. However, no new peak corresponding to the PTZ^*δ*+^-C_60_^*δ*−^ CT state in the visible or near-IR regions was apparent, suggesting low molar absorptivity of such transitions if they existed.

Among the studied compounds, PTZ-PTZ having only PTZs was found to be fluorescent with a peak maximum at 526 nm. Lifetime measurements resulted in a *τ* value of 6.0 ns. Other compounds carrying TCBD/DCNQ and C_60_ were found to be weakly or nonfluorescent. CT-type emission was observed for PTZ-DCNQ-PTZ with a peak maxima at 648 nm, while for PTZ-TCBD-PTZ, a very weak signal at 576 nm was also observed. For PTZ-PTZ-C_60_, PTZ-TCBD-PTZ-C_60_ and PTZ-DCNQ-PTZ-C_60_ carrying an additional C_60_, no emission (*Φ*_f_ < 10^−5^) was noted.

### Electrochemical and spectro-electrochemical studies

**Fig. 3:**
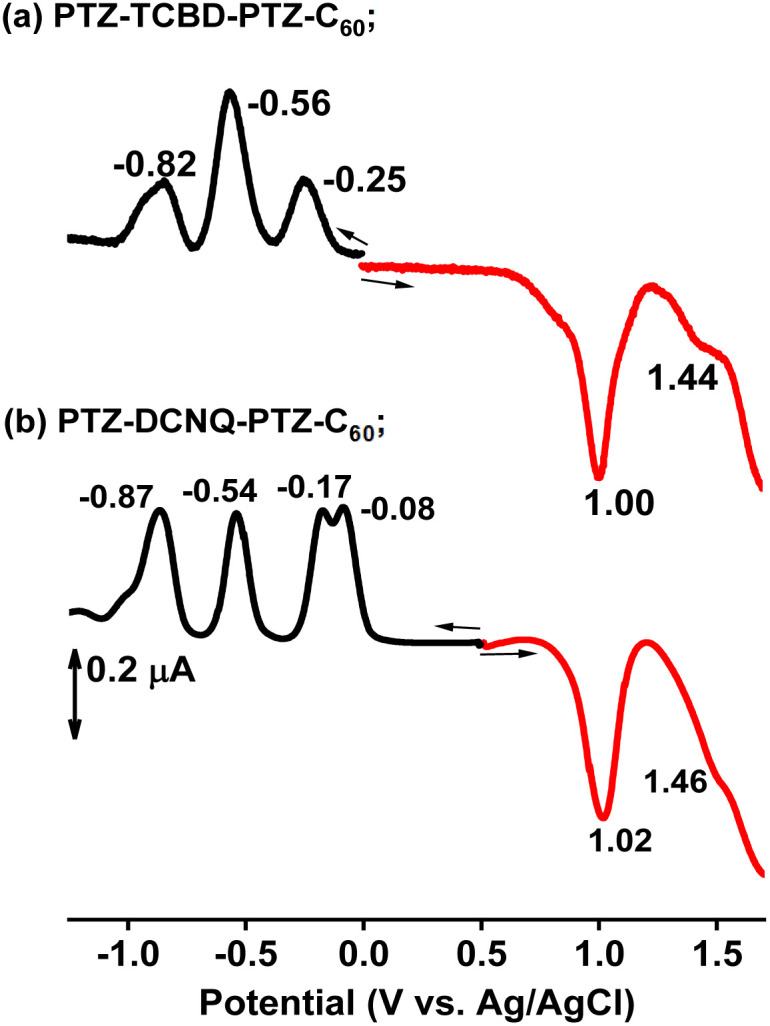
DPVs of compounds (a) PTZ-TCBD-PTZ-C_60_ and (b) PTZ-DCNQ-PTZ-C_60_ in DCB containing 0.1 M (TBA)ClO_4_ (tetrabutylammonium perchlorate).

Electrochemical redox potentials are essential in establishing electron transfer mechanisms, especially for complex systems investigated here. Fig. 3 shows the differential pulse voltammograms (DPVs) of compounds PTZ-PTZ-C_60_, PTZ-TCBD-PTZ-C_60_ and PTZ-DCNQ-PTZ-C_60_, while voltammograms of other systems are shown in Fig. S19.† The control compounds shown in Fig. 1 helped assign the site of electron transfer in these systems. For compound PTZ-TCBD-PTZ-C_60_, the PTZ oxidation was located at 1.0 V *vs.* Ag/AgCl while the TCBD reductions were located at −0.25 and −0.56 V. For this compound, C_60_ reduction overlapped with the second reduction of TCBD with a peak at −0.56 V. In the case of PTZ-DCNQ-PTZ-C_60_, while PTZ oxidation was almost invariant and appeared at 1.02 V, the DCNQ reductions appeared at −0.08 and −0.17 V while C_60_-centered reductions were at −0.54 and −0.87 V. Two key observations were borne from this study, *viz.*, (i) facile reduction of TCBD and DCNQ compared to C_60_, with DCNQ being a better electron acceptor than TCBD (*E*_red_ order: DCNQ < TCBD < C_60_), and (ii) small electrochemical redox gap (1.25 V for PTZ-TCBD-PTZ-C_60_ and 1.10 V for PTZ-DCNQ-PTZ-C_60_). Spectro-electrochemical results were also performed to characterize the oxidized and reduced species spectrally, and are summarized in the ESI (see Fig. S20 and S21).†

### Computational studies

**Fig. 4:**
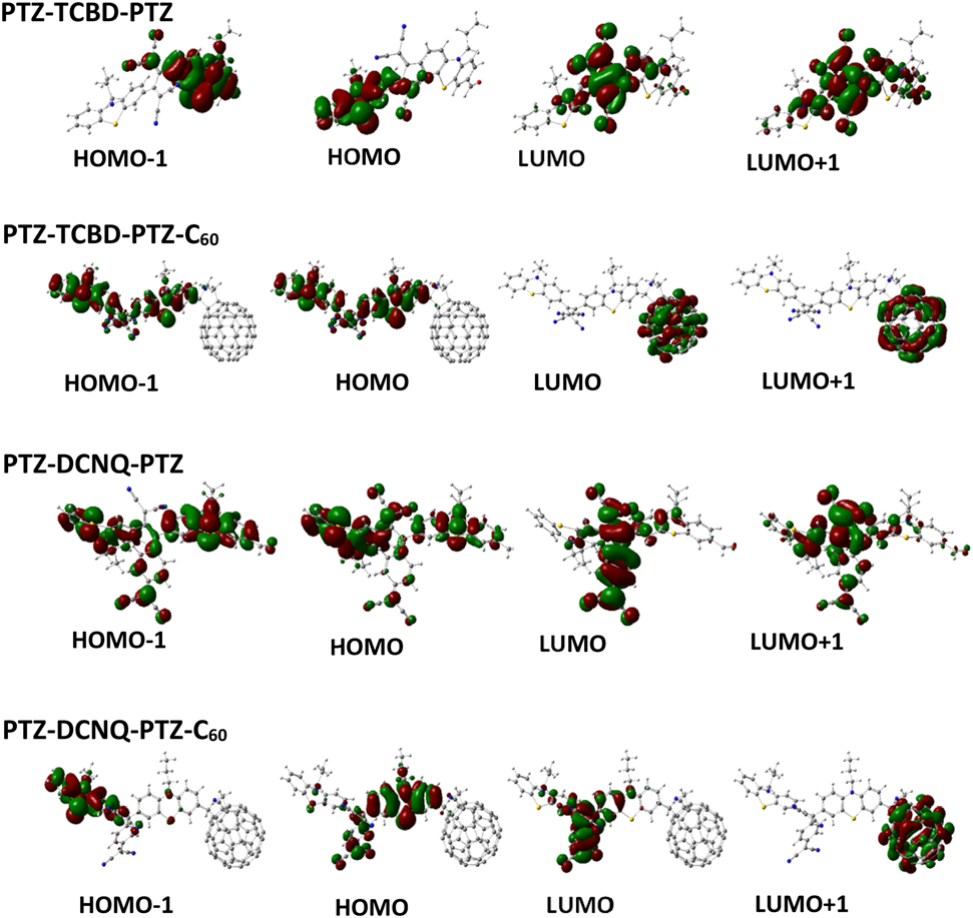
Frontier HOMOs and LUMOs of the indicated compounds on B3LYP/6-311+G(d,p) optimized structures in the gas phase.

Computational studies, particularly density functional theory (DFT) and time-dependent DFT (TD-DFT), play a crucial role in elucidating the entities of multi-modular systems that participate in forming charge transfer (CT) states, both in the ground and excited states. All geometry optimizations were carried out using DFT at the B3LYP hybrid functional and 6-311+G(d,p) basis set with one set of diffuse s and p functions on heavy atoms, as implemented in Gaussian 16. Optimizations were carried out in the gas phase without the use of an implicit or explicit solvation model. Here, the default Gaussian convergence criteria were employed with default convergence thresholds. The excited state properties were computed using TD-DFT at the same level of theory, with the first 10 singlet states evaluated with the TD keyword TD = (Singlets, NStates = 10, Root = 1). The electronic density of the target excited state was retained for analysis (density = current).^25,26^ The HOMOs and LUMOs of PTZ-PTZ-C_60_ and PTZ-PTZ on their optimized structures are shown in Fig. S22 in ESI.† The electron density of the HOMO spread over both PTZ entities while the LUMO was focused on the C_60_ in the case of PTZ-PTZ-C_60_. Fig. 4 compares the frontier orbitals of PTZ-TCBD-PTZ*vs.*PTZ-TCBD-PTZ-C_60_ and PTZ-DCNQ-PTZ*vs.*PTZ-DCNQ-PTZ-C_60_, that is, TCBD/DCNQ carrying systems with and without C_60_. As predicted, in the case of PTZ-TCBD-PTZ, HOMOs on the PTZ and LUMO on the TCBD, and in the case of PTZ-DCNQ-PTZ, most HOMOs on PTZ and LUMOs on DCNQ were observed. Interestingly, for PTZ-TCBD-PTZ-C_60_ having both TCBD and C_60_ acceptors, HOMOs were spread over the PTZ-TCBD-PTZ part (little contributions on TCBD) of the molecule, while LUMO and LUMO+1 were on the C_60_, suggesting in this molecular design, C_60_ would be the ultimate electron acceptor although TCBD had lower reduction potential. In the case of PTZ-DCNQ-PTZ-C_60_, HOMOs are on PTZ-DCNQ (little contributions on DCNQ), and the first LUMO on DCNQ and the second one on C_60_ was witnessed. This suggests that even in PTZ-DCNQ-PTZ-C_60_, which has the most potent electron acceptor, DCNQ, the final charge could reside on C_60_. To support this, additional calculations on control TCBD-Ph and DCNQ-Ph (electron acceptors without the donor entity) were performed, and their orbital distribution and LUMO energies were compared, as shown in Fig. S28.† Higher energies of LUMO in PTZ-TCBD-PTZ and PTZ-DCNQ-PTZ suggest them as poor electron acceptors compared to the controls. Should these predictions hold strong, the TD-DFT studies should show the location of the final radical cation and anion in photoinduced electron transfer.

**Fig. 5:**
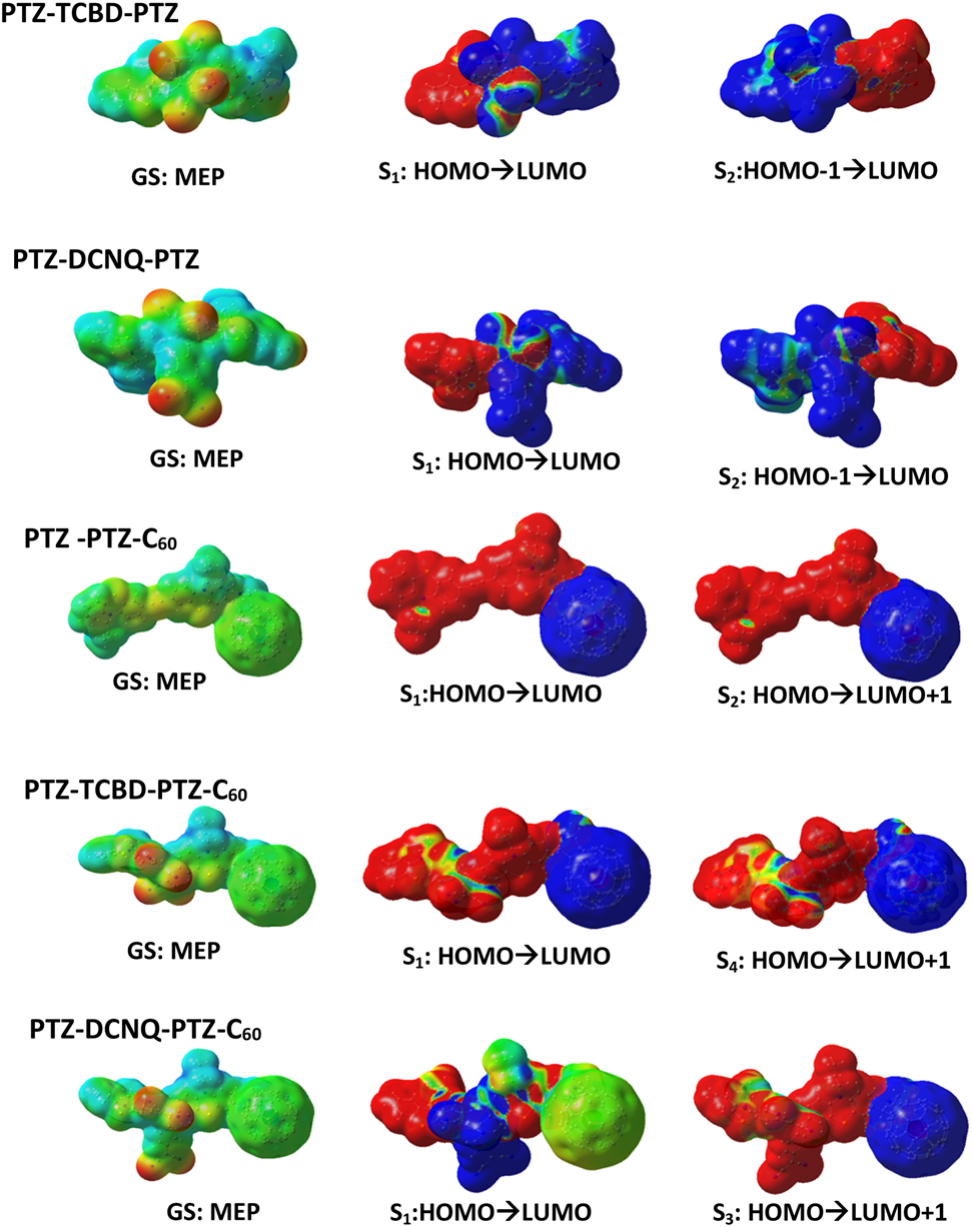
Illustration of MEPs revealing ground state polarization and CS from different excited states for the investigated compounds. Electrostatic potential surface: red = electron-rich regions, blue = electron-poor regions (scale – 5.000 × 10^−2^ to 5.000 × 10^2^ V).

Fig. 5 and S23† show the molecular electrostatic potential maps (MEPs) and the excited charge transfer locations supporting the above-narrated charge polarization in the ground state and charge separation (CS) from the singlet excited states. For PTZ-TCBD-PTZ, the formation of PTZ˙^+^-(TCBD-PTZ)˙^−^ from S_1_ state and (PTZ-TCBD)˙^−^-PTZ˙^+^ from S_2_ state; and for PTZ-DCNQ-PTZ, the formation of PTZ˙^+^-(DCNQ-PTZ)˙^−^ from S_1_ state and (PTZ-DCNQ)˙^−^-PTZ˙^+^ from S_2_ state is observed. The PTZ entities are alternatively involved with TCBD/DCNQ to form the CS products. Expectedly, for PTZ-PTZ-C_60_, having no TCBD or DCNQ but only C_60_, the formation of (PTZ-PTZ)˙^+^-C_60_˙^−^ from both the first and second excited states is clear. On the contrary, for PTZ-TCBD-PTZ-C_60_, having both TCBD and C_60_, the formation of (PTZ-TCBD-PTZ)˙^+^-C_60_˙^−^ from S_1_ and S_4_ states (with little electron density on TCBD) was observed. Similarly, for PTZ-DCNQ-PTZ-C_60_ having both DCNQ and C_60_, the CS products involved DCNQ from the S_1_ state, while from the S_3_ state (PTZ-DCNQ-PTZ)˙^+^-C_60_˙^−^ was the expected product. In summary, the involvement of terminal C_60_ in CS reaction, even though it is not the most potent electron acceptor compared to TCBD or DCNQ, is borne from this study.

### Energy level diagram

**Fig. 6:**
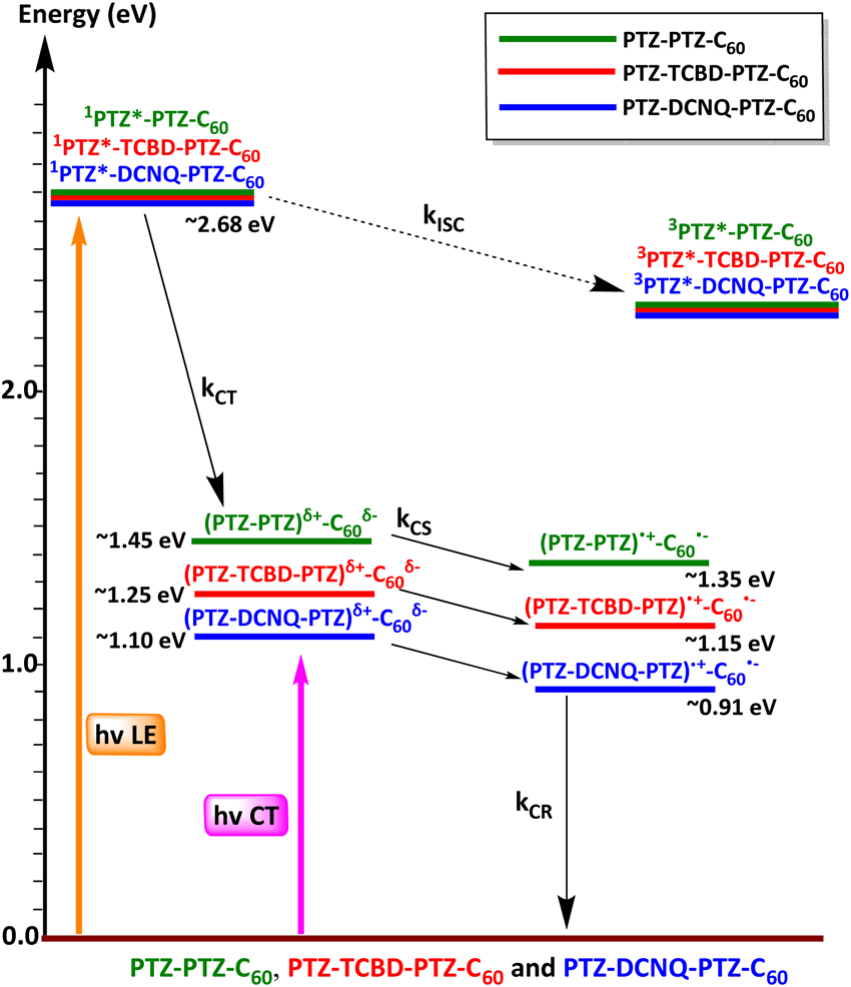
Energy level diagram depicts the different photochemical events occurring in PTZ-PTZ-C_60_, PTZ-TCBD-PTZ-C_60_ and PTZ-DCNQ-PTZ-C_60_ in 1,2-dichlorobenzene. Energies of different states were evaluated from spectral and electrochemical studies.^28^ The solid arrows indicate major photo processes, and the dashed arrow indicates minor photo processes. Abbreviations: CT = charge transfer, CS = charge separation, ISC = intersystem crossing, CR = charge recombination.^27^§

Fig. 6 shows an energy level diagram constructed based on the above findings.^27^§§Gibbs free-energy change associated with excited state charge separation (CS) and dark charge recombination (CR) was estimated according to eqn (1)–(3).^27^
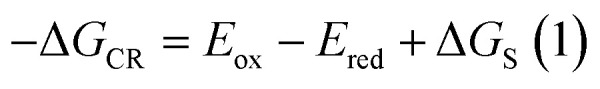

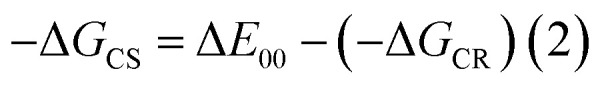
where Δ*E*_00_ corresponds to the singlet state energy of 1. The term Δ*G*_S_ refers to electrostatic energy calculated according to the dielectric continuum model (see eqn (3)). The *E*_ox_ and *E*_red_ represent the oxidation potential and the first reduction potential, respectively.
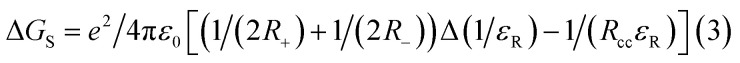
The symbols *ε*_0_ and *ε*_R_ represent the vacuum permittivity and dielectric constant of DCB used for photochemical and electro-chemical studies. *R*_CC_ is the center-to-center distance between donor and acceptor entities from the computed structures. The energy of charge transfer was calculated from the peak maxima of the charge transfer peak. In the case of both PTZ-TCBD-PTZ-C_60_ and PTZ-DCNQ-PTZ-C_60_, excitation from the LE and CT states is expected to result in initial CT and final CS products with either full or most of the radical anion residing on terminal C_60_. This should be much clearer for the CS product than the initial CT product, which revealed contributions from central TCBD and DCNQ. Intrigued by these unexpected findings, femtosecond transient absorption (fs-TA) studies were performed to gather the experimental proof, as discussed below.

### Femtosecond transient absorption data

When identifying electron transfer products involving C_60_, the near-IR peak in the ∼1000 nm range nm with a moderate absorptivity corresponding to C_60_˙^−^ plays an essential role in providing the spectral identity and evaluating the kinetics of such events. In the complex systems studied here, comparing transient data of control compounds (systems without C_60_) secured under identical conditions also plays a significant role. In the present study, we have utilized compounds PTZ-TCBD-PTZ and PTZ-DCNQ-PTZ, having no C_60_ as controls for compounds PTZ-TCBD-PTZ-C_60_ and PTZ-DCNQ-PTZ-C_60_. In addition, the compounds are excited at the CT peak (555 for PTZ-TCBD-PTZ-C_60_ and PTZ-TCBD-PTZ, and 665 nm for PTZ-DCNQ-PTZ-C_60_ and PTZ-DCNQ-PTZ) as C_60_ has little or no absorption beyond 525 nm, avoiding simultaneous excitation and complicating the process.

**Fig. 7:**
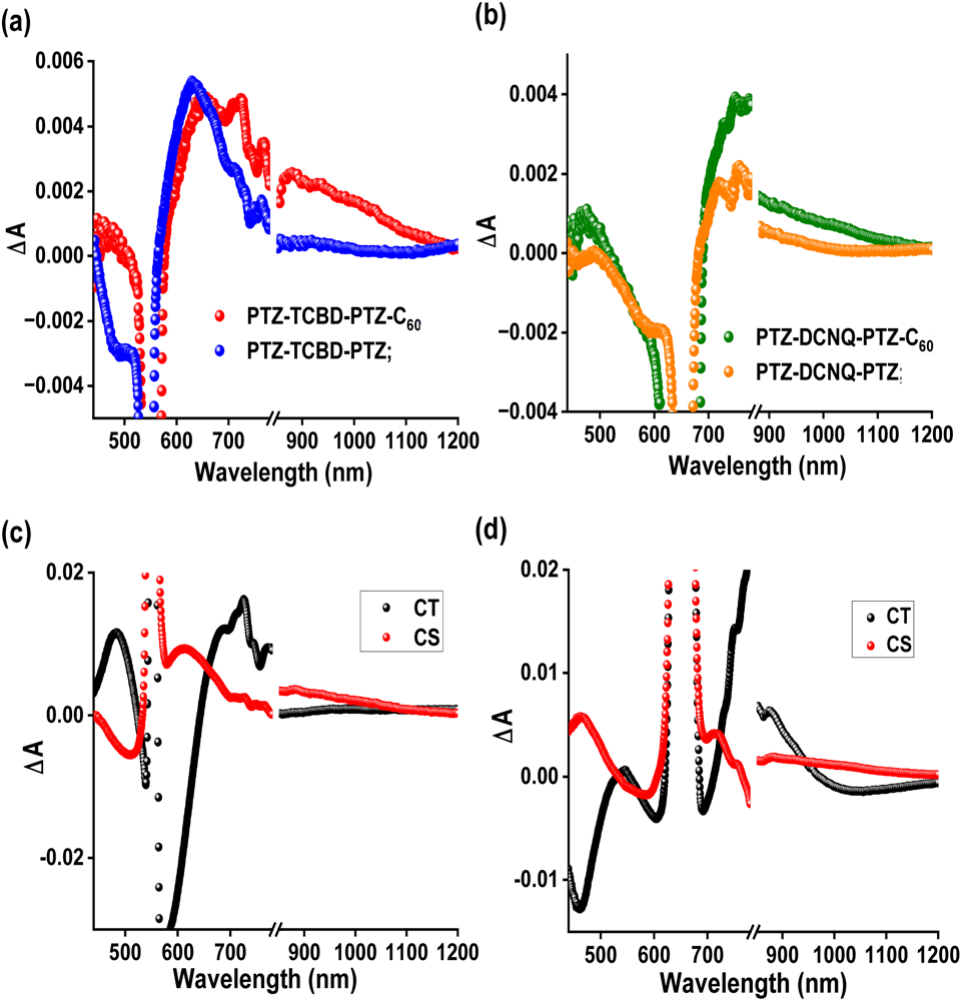
Fs-TA spectrum (a) at a delay time of 63.98 ps for PTZ-TCBD-PTZ-C_60_ (red) and PTZ-TCBD-PTZ (blue), and (b) at a delay of 53.60 ps for PTZ-DCNQ-PTZ (orange) and PTZ-DCNQ-PTZ-C_60_ (green) in DCB. Compounds in (a) were excited at 555 nm and (b) at 665 nm, corresponding to their charge transfer bands. The decay-associated spectra (DAS) from GloTarAn analysis for (c) PTZ-TCBD-PTZ-C_60_ and (d) PTZ-DCNQ-PTZ-C_60_ are shown below.

Fig. 7a shows the fs-TA spectra recorded for PTZ-TCBD-PTZ-C_60_ and PTZ-TCBD-PTZ at a delay time of 63.98 ps. Clear peaks in the 440 and 650–720 nm range corresponding to the cation radical of PTZ-TCBD-PTZ (see spectroelectrochemical results given in Fig. S20 and S21†) and 1000 nm range for anion radical of C_60_ (red lines) were obvious. In the near-IR region, the control compound PTZ-TCBD-PTZ revealed no peaks (blue line), confirming that the observed broad peak is indeed due to C_60_˙^−^ (see Fig. S24† for complete transient data on PTZ-TCBD-PTZ-C_60_ and PTZ-TCBD-PTZ). Similar observations were made for PTZ-DCNQ-PTZ-C_60_ and PTZ-DCNQ-PTZ; *i.e.*, absorption in the 1000 nm range for PTZ-DCNQ-PTZ-C_60_ (green) and peaks expected for cation radical of PTZ-DCNQ-PTZ in the visible range (Fig. 7b). Such transient peaks in the near-IR region were absent for PTZ-DCNQ-PTZ (see Fig. S25† for complete transient data of PTZ-DCNQ-PTZ-C_60_ and PTZ-DCNQ-PTZ). These results conclusively support the involvement of C_60_ in the CS process for both PTZ-TCBD-PTZ-C_60_ and PTZ-DCNQ-PTZ-C_60_. The transient data were subjected to GloTarAn analysis^28^ to deconvolute the spectra and to identify the corresponding kinetic parameters. The decay-associated spectra (DAS) are shown in Fig. 6c and d. A two-component fit corresponding to the CT and CS processes was satisfactory. An average lifetime of 21.7 and 279.3 ps corresponding to the CT and CS events of PTZ-TCBD-PTZ-C_60_, and 29.1 and 180.2 ps corresponding to the CT and CS events of PTZ-DCNQ-PTZ-C_60_ arrived (see ESI Fig. S26 and S27† for complete GloTarAn analysis data), confirming the theoretical expectations supporting experimental evidence.

## Conclusions

To summarize, supported by time-dependent DFT theoretical calculations, for the first time, we have demonstrated the superior electron acceptor properties of C_60_ in the presence of stronger electron acceptors in multi-modular configurations. The multi-redox-carrying molecular systems designed and synthesized as part of this key investigation had unique features that differentiated them from simple donor–acceptor systems in literature, and the findings provided unprecedented results. Studies show that the electron–acceptor character of both TCBD and DCNQ sandwiches between two electron–donor phenothiazine entities is diminished, making the terminal C_60_ the ultimate electron acceptor. These results make the wide-band capturing push–pull systems studied here useful for various energy harvesting and optoelectronic applications (*e.g.*, photo(electro)catalysis and optoelectronics, including OLEDs). Further studies are in progress to unravel the full potential of this class of molecular systems, and the reasoning behind this unprecedented phenomenon, which may be a small reorganization energy or structure/geometry factor, is in progress in our laboratories.

## Data availability

Additional data and figures are available in the ESI.†

## Author contributions

Methodology: synthesis, PKG and RM; computational studies, photo – and electrochemistry, CVI; funding acquisition, FD; FD and RM conceived the project and supervised the work. All authors have approved the published version of the manuscript.

## Conflicts of interest

There are no conflicts to declare.

## Supplementary Material

SC-OLF-D5SC02950C-s001
