# Supplementary material for: Strong acceptor incorporated phenothiazine-C60 multi-redox push–pull conjugates: demonstration of C60's superior electron acceptor characteristics
Source: Chem Sci. 2025 May 29;16(26):12122–8. doi: 10.1039/d5sc02950c (PMC12143295; doi:10.1039/d5sc02950c)
Supplement: SC-016-D5SC02950C-s001 [file SC-016-D5SC02950C-s001.pdf]

Supplementary Information (SI) for Chemical Science.
This journal is © The Royal Society of Chemistry 2025

# Electronic Supplemental Information

## Strong acceptor incorporated phenothiazine-$C_{60}$ multi-redox push-pull conjugates: Demonstration of $C_{60}$'s superior electron acceptor characteristics

Pankaj K. Gupta, ‡[a] Chamari V. Ileperuma, ‡[b] Rajneesh Misra,*[a] and Francis D'Souza*[b]

[a] Department of Chemistry, Indian Institute of Technology Indore, Indore 453552, India, E-mail: rajneeshmisra@iiti.ac.in

[b] Department of Chemistry, University of North Texas at Denton, 1155 Union Circle, #305070, Denton, TX 76203-5017, USA, E-mail: Francis.DSouza@unt.edu

‡ Equal contributions

## Table of contents


| | | | |
|---|---|---|---|
| 1. | **Experimental details** | | S3-S7 |
| 2. | **Figure S1.** $^{1}$H-NMR of **PTZ-PTZ** | | S8 |
| 3. | **Figure S2.** $^{13}$C-NMR of **PTZ-PTZ** | | S8 |
| 4. | **Figure S3**. HRMS of **PTZ-PTZ** | | S9 |
| 5. | **Figure S4.** $^{1}$H-NMR of **PTZ-TCBD-PTZ** | | S10 |
| 6. | **Figure S5.** $^{13}$C-NMR of **PTZ-TCBD-PTZ** | | S10 |
| 7. | **Figure S6.** HRMS of **PTZ-TCBD-PTZ** | | S11 |
| 8. | **Figure S7.** $^{1}$H-NMR of **PTZ-DCNQ-PTZ** | | S12 |
| 9. | **Figure S8.** $^{13}$C-NMR of **PTZ-DCNQ-PTZ** | | S12 |
| 10. | **Figure S9.** HRMS of **PTZ-DCNQ-PTZ** | | S13 |
| 11. | **Figure S10.** $^{1}$H-NMR of **PTZ-PTZ-$C_{60}$** | | S14 |
| 12. | **Figure S11.** $^{13}$C-NMR of **PTZ-PTZ-$C_{60}$** | | S14 |
| 13. | **Figure S12.** HRMS of **PTZ-PTZ-$C_{60}$** | | S15 |
| 14. | **Figure S13.** $^{1}$H-NMR of **PTZ-TCBD-PTZ-$C_{60}$** | | S16 |
| 15. | **Figure S14.** $^{13}$C-NMR of **PTZ-TCBD-PTZ-$C_{60}$** | | S16 |
| 16. | **Figure S15.** MALDI of **PTZ-TCBD-PTZ-$C_{60}$** | | S17 |
| 17. | **Figure S16.** $^{1}$H-NMR of **PTZ-DCNQ-PTZ-$C_{60}$** | | S17 |
| 18. | **Figure S17.** $^{13}$C-NMR of **PTZ-DCNQ-PTZ-$C_{60}$** | | S18 |
| 19. | **Figure S18.** MALDI of **PTZ-DCNQ-PTZ-$C_{60}$** | | S18 |
| 20. | **Figure S19.** Differential pulse voltammograms (DPV) and cyclic voltammograms (CV) of compounds **PTZ-TCBD-PTZ-$C_{60}$, PTZ-DCNQ-PTZ-$C_{60}$, PTZ-PTZ , PTZ-TCBD-PTZ, PTZ-DCNQ-PTZ** in dichlorobenzene with 0.1 M (TBA)$ClO_4$ | | S19 |
| 21. | **Table S1.** Redox potentials, free-energy change for charge transfer ($\Delta G_{CT}$), separation ($\Delta G_{CS}$) and recombination ($\Delta G_{CR}$) for the investigated compounds in 1,2-dichlorobenzene | | S19 |
| 22. | **Figure S20.** Spectral changes observed during first oxidation and first reduction of **PTZ-TCBD-PTZ** and **PTZ-TCBD-PTZ-$C_{60}$** in dichlorobenzene with 0.2 M (TBA)$ClO_4$ | | S20 |
| 23. | **Figure S21.** Spectral changes observed during first oxidation and first reduction of **PTZ-DCNQ-PTZ** and **PTZ-DCNQ-PTZ-$C_{60}$** in dichlorobenzene with 0.2 M (TBA)$ClO_4$ | | S21 |
| 24. | **Figure S22.** Frontier HOMOs and LUMOs and their energies of the compound **PTZ-PTZ** and **PTZ-PTZ-$C_{60}$** in gas phase | | S22 |
| 25. | **Figure S23.** Electrostatic Potential Surfaces and the charge transfer locations for the investigated compounds on B3LYP/6-311+G(d,p) optimized structures in the gas phase. | | S23 |
| 26. | **Figure S24**. Fs-TA Spectra of **PTZ-TCBD-PTZ-$C_{60}$** and **PTZ-TCBD-PTZ** in 1,2-dichlorobenzene (λex = 555 nm) | | S24 |
| 27. | **Figure S25.** Fs-TA Spectra of **PTZ-DCNQ-PTZ-$C_{60}$** and **PTZ-DCNQ-PTZ** in 1,2-dichlorobenzene (λex = 665 nm) | | S24 |
| 28. | **Figure S26.** Fs-TA Spectra, decay-associated spectra (DAS), and population kinetics from GloTarAn analysis of **PTZ-TCBD-PTZ-$C_{60}$** in 1,2-dichlorobenzene (λex =665 nm). | | S25 |
| 29. | **Figure S27.** Fs-TA Spectra, decay-associated spectra (DAS), and population kinetics from GloTarAn analysis of **PTZ-DCNQ-PTZ-$C_{60}$** in 1,2-dichlorobenzene (λex =665 nm). | | S26 |
| 30 | **Figure S28.** Comparison of HOMO and LUMO orbital distribution and energies of TCBD-Ph and PTZ-TCBD-PTZ, and DCNQ-Ph and PTZ-DCNQ-PTZ. | | S27 |

**Experimental details:**

The intermediates **Br-PTZ-CHO** and **PTZ** were synthesized according to the reported procedures.[1-3]

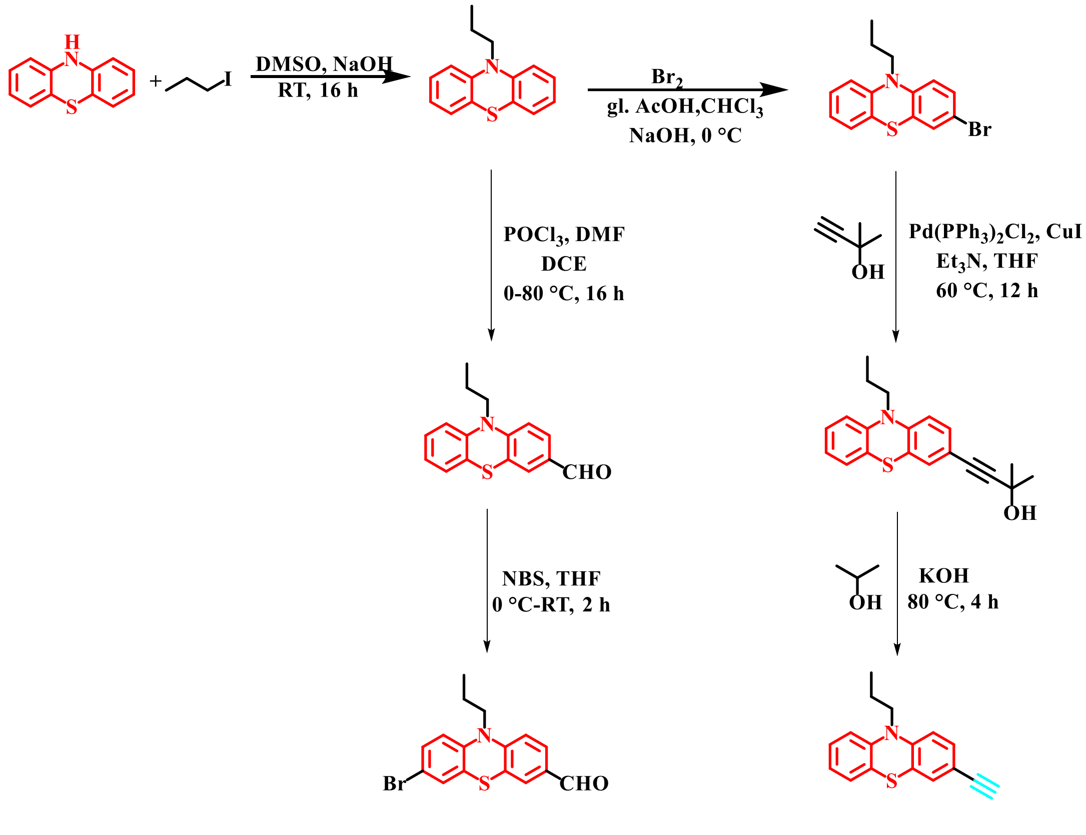


**Br-PTZ-CHO** **PTZ**

**Scheme S1**. Synthetic route for intermediates Br-PTZ-CHO and PTZ.

**Synthesis of 7-bromo-10-propyl-10*H*-phenothiazine-3-carbaldehyde (Br-PTZ-CHO)**

In a 250 mL rounded bottom flask, Phenothiazine (5.0g, 25.1 mmol) and propyl iodide (5.5g, 3.18mL, 32.6 mmol) were dissolved in 50mL DMSO and stirred for 30min at room temperature. Sodium hydroxide (2.8g, 50 mmol) was slowly added and stirred for overnight at room temperature. The reaction mixture was poured into water and extracted with dichloromethane. The organic layer was separated and dried over anhydrous sodium sulfate. The product was purified using column chromatography with hexane. The product was obtained as white solid with 97% yield. After that, the white solid compound (5 g, 1.0 eq) and dry DMF (4.0 eq) was dissolved in 1,2-dichloroethane then phosphorous oxychloride (4.0 eq) was added slowly at 0 °C in an ice water bath. The mixture was heated to reflux overnight and was quenched with water and extracted three times with dichloromethane. The combined organic fraction was washed with brine and dried over $Na_2SO_4$. The solvent was removed under reduced pressure and the residue was purified by column chromatography using silica gel and n-hexane/dichloromethane (1/1; v/v) as the eluent to give a yellow solid with 80% yield. After that, the NBS (1.1 eq) was added in one portion to the solution of 3 (1.5 g, 1.0 eq) in THF (50 mL) at 0 °C. The mixture was allowed to room temperature and continued

stirring for 2 h. Then the reaction was quenched by addition of water (50 mL) and extracted with DCM. The collected organic layer was evaporated under vacuum and the residue was purified by column chromatography to give the product a yellow solid compound **Br-PTZ-CHO** with 90% yield.

**Synthesis of 3-ethynyl-10-propyl-10*H*-phenothiazine (PTZ)**

In a 100 mL rounded bottom flask, 3-bromo-10-propyl-10*H*-phenothiazine (2.3 g, 5.7 mmol) and 2-methylbut-3-yn-2-ol (969 mg, 11.52 mmol) were purged under nitrogen and bis(triphenylphosphine)palladium(II)dichloride (50 mg) and copper(I) iodide (2 - 3 mg) were added in anhydrous triethylamine (15 mL) and tetrahydrofuran (15 mL), the reaction mixture was stirred and refluxed for 12 h. Then the solvent was evaporated under a vacuum. The residue was dissolved in dichloromethane, and this solution was washed with water for several times, dried with anhydrous $Na_2SO_4$, and filtered. The crude was purified by column chromatography using dichloromethane-hexane (1/3 v/v) as an eluent to give a protected product. After deprotection of this protected product with the help of KOH (2 equivalent) and Isopropyl alcohol (20 mL), the reaction mixture was stirred and refluxed for 4 h at 80 °C. Then the solvent was evaporated under a vacuum. The residue was dissolved in dichloromethane, and this solution was washed with water for several times, dried with anhydrous $Na_2SO4$, and filtered. The crude was purified by column chromatography using hexane as eluent to give a yellowish oily compound **PTZ** formed with 65% yield.

**General methods**

The chemicals were used as received unless otherwise indicated. All oxygen or moisture-sensitive reactions were performed under a nitrogen/argon atmosphere. $^1$H NMR (400 and 500 MHz) and $^{13}$C NMR (100 and 125 MHz) spectra were recorded on Bruker 400 and 500 MHz FT-NMR spectrometers at room temperature. $^1$H NMR chemical shifts are reported in parts per million (ppm) relative to the solvent residual peak ($CDCl_3$, 7.26 ppm). Multiplicities are given as s (singlet), d (doublet), t (triplet) and m (multiplet) and the coupling constants, *J*, are given in hertz. $^{13}$C NMR chemical shifts are reported relative to the solvent residual peak ($CDCl_3$, 77.0 ppm). UV-visible absorption spectra were recorded on a PerkinElmer Lambda 35 instrument. HRMS was recorded with an Agilent 6545A Q-TOF mass spectrometer and on a Bruker-Daltonics micrOTOF-Q II mass spectrometer and MALDI-TOF mass used to measure the mass of the compounds. The voltammograms were recorded on a GAMRY Reference 620 Potentiostat in 1,2-dichlorobenzene solvent and 0.1 M (TBA)$ClO_4$ as the supporting electrolyte. The electrodes used were glassy carbon as a working electrode, Pt wire as a counter electrode and Ag/AgCl as a reference electrode, the scan rate was 100 mV $s^{-1}$ for cyclic voltammetry.

**Synthesis of PTZ-PTZ**

In a 100 mL round-bottomed flask, 3-ethynyl-10-propyl-10*H*-phenothiazine **PTZ** (550 mg, 2.072 mmol) and 7-bromo-10-propyl-10*H*-phenothiazine-3-carbaldehyde **Br-PTZ-CHO** (761 mg, 2.279 mmol) were purged under nitrogen and bis(triphenylphosphine)palladium(II) dichloride (100 mg) and copper(I) iodide (4 - 5 mg) were added in anhydrous

triethylamine (20 mL) and tetrahydrofuran (20 mL), the reaction mixture was stirred and refluxed for 12 h. Then the solvent was evaporated under a vacuum. The residue was dissolved in dichloromethane, and this solution was washed with water for several times, dried with anhydrous $Na_2SO_4$, and filtered it. The crude was purified by column chromatography using dichloromethane-hexane (1/2 v/v) as eluent to get the desired compound **PTZ-PTZ** as a yellow color solid with 87 % yield. $^1H$ NMR (500 MHz, chloroform-d) d = 9.80 (s, 1 H), 7.64 (dd, *J* = 1.8, 8.5 Hz, 1 H), 7.57 (d, *J* = 1.7 Hz, 1 H), 7.29 - 7.27 (m, 1 H), 7.26 - 7.21 (m, 3 H), 7.14 (s, 1 H), 7.11 (d, *J* = 7.5 Hz, 1 H), 6.94 - 6.91 (m, 1 H), 6.90 (d, *J* = 8.4 Hz, 1 H), 6.85 (d, *J* = 8.1 Hz, 1 H), 6.80 (d, *J* = 8.5 Hz, 1 H), 6.78 (d, *J* = 8.4 Hz, 1 H), 3.89 - 3.83 (m, 2 H), 3.83 - 3.79 (m, 2 H), 1.86 (d, *J* = 7.5 Hz, 2 H), 1.84 - 1.80 (m, 2 H), 1.06 - 1.02 (m, 3 H), 1.02 - 0.99 (m, 3 H); $^{13}C$ NMR (125 MHz, chloroform-d) d = 190.0, 150.1, 145.3, 144.6, 143.1, 131.3, 130.8, 130.6, 130.1, 130.0, 128.5, 127.5, 127.3, 124.8, 124.5, 124.2, 123.8, 122.7, 118.7, 116.9, 115.6, 115.5, 115.1, 115.0, 89.4, 88.1, 49.8, 49.3, 20.1, 20.0, 11.3, 11.2, 11.1; HRMS (ESI-TOF) m/z calculated for $C_{33}H_{28}N_2OS_2$ 532.1638 $[M]^+$, measured 532.1274 $[M]^+$.

## Synthesis of PTZ-TCBD-PTZ

In a 100 mL round-bottomed flask, TCNE (40 mg, 0.3097 mmol) was added to a solution of **PTZ-PTZ** (150 mg, 0.2815 mmol) in dichloromethane (20 mL) under an argon atmosphere. The reaction mixture was stirred at RT for 24 h. The solvent was evaporated under vacuum, and the product was purified by column chromatography with DCM/hexane (2:1, v/v) as the eluent to get the desired compound **PTZ-TCBD-PTZ** as a dark color solid with 80 % yield. $^1H$ NMR (500MHz, chloroform-d) d = 9.83 (s, 1 H), 7.71 - 7.68 (m, 2 H), 7.68 - 7.65 (m, 1 H), 7.55 (d, *J* = 1.5 Hz, 1 H), 7.34 (d, *J* = 2.4 Hz, 1 H), 7.32 (d, *J* = 2.3 Hz, 1 H), 7.18 (s, 1 H), 7.08 - 7.05 (m, 1 H), 7.01 (d, *J* = 7.3 Hz, 1 H), 6.96 (d, *J* = 8.5 Hz, 1 H), 6.93 (d, *J* = 9.0 Hz, 1 H), 6.88 (t, *J* = 8.7 Hz, 2 H), 3.90 (d, *J* = 7.3 Hz, 2 H), 3.88 - 3.84 (m, 2 H), 1.89 (d, *J* = 7.3 Hz, 2 H), 1.87 - 1.82 (m, 2 H), 1.07 (d, *J* = 7.3 Hz, 3 H), 1.04 (d, *J* = 7.5 Hz, 3 H); $^{13}C$ NMR (100 MHz, chloroform-d) d = 189.7, 164.4, 163.6, 151.3, 149.5, 147.6, 141.9, 132.6, 130.5, 130.4, 130.3, 128.5, 128.0, 127.8, 127.7, 127.6, 125.9, 125.6, 125.3, 124.6, 124.4, 123.6, 122.6, 116.3, 115.9, 115.1, 112.9, 112.6, 112.2, 111.8, 82.7, 80.6, 53.4, 50.4, 50.0, 34.7, 31.6, 25.3, 22.7, 20.1, 20.0, 14.1, 11.1, 11.0; HRMS (ESI-TOF) m/z calculated for $C_{39}H_{28}N_6OS_2$ + Na 683.1658 $[M+Na]^+$, measured 683.4354 $[M+Na]^+$.

## Synthesis of PTZ-DCNQ-PTZ

In a 100 mL round-bottomed flask, TCNQ (43 mg, 0.2065 mmol) was added to a solution of **PTZ-PTZ** (100 mg, 0.1877 mmol) in dichloroethane (20 mL) under an argon atmosphere. The reaction mixture was stirred at 60 °C for 36 hours. The solvent was evaporated under vacuum, and the product was purified by column chromatography with DCM/hexane (3:1, v/v) as the eluent to get the desired compound **PTZ-DCNQ-PTZ** as a dark color solid with 60 % yield. $^1H$ NMR (500 MHz, chloroform-d) d = 9.80 (s, 1 H), 7.64 (d, *J* = 8.1 Hz, 1 H), 7.57 (d, *J* = 8.7 Hz, 1 H), 7.52 (s, 1 H), 7.47 (d, *J* = 9.6 Hz, 1 H), 7.32 - 7.28 (m, 2 H), 7.22 (d, *J* = 9.5 Hz, 1 H), 7.17 - 7.11 (m, 2 H), 7.08 (d, *J* = 7.5 Hz, 1 H), 6.99 - 6.96 (m, 2 H), 6.95 - 6.90 (m, 2 H), 6.88 - 6.83 (m, 3 H), 3.89 - 3.80 (m, 4 H), 1.84 (dd, *J* = 7.2, 14.4 Hz, 4 H),

1.06 - 1.01 (m, 6 H); $^{13}$C NMR (100 MHz, chloroform-d) d = 189.6, 168.3, 153.9, 148.9, 148.8, 148.7, 147.8, 142.8, 134.7, 133.9, 133.5, 132.4, 131.8, 130.3, 129.2, 128.9, 128.4, 128.2, 127.8, 127.6, 126.4, 126.2, 125.7, 125.1, 123.9, 123.7, 123.0, 116.0, 115.8, 115.8, 115.4, 113.7, 113.2, 112.7, 83.6, 76.1, 50.3, 49.8, 29.7, 26.9, 20.1, 20.0, 14.1, 11.2, 11.1; HRMS (ESI-TOF) m/z calculated for $C_{45}H_{32}N_6OS_2$ + Na 759.1971 [M+Na]$^+$, measured 759.2002 [M+Na]$^+$.

### Synthesis of PTZ-PTZ-$C_{60}$

In a 100 mL round-bottomed flask, compound **PTZ-PTZ** (90 mg, 0.1689 mmol), $C_{60}$-fullerene (243 mg, 0.3378 mmol) and *N*-methylglycine (120 mg, 1.352 mmol) were dissolved in toluene (20 mL) and refluxed for 36 h. After that, the reaction mixture was extracted with DCM, washed with brine solution and dried over anhydrous sodium sulfate. The solvent was evaporated under vacuum, and the product was purified by column chromatography with DCM/hexane (1:1, v/v) as the eluent to get the desired compound **PTZ-PTZ-$C_{60}$** as a dark color solid with 40 % yield. $^1$H NMR (500 MHz, chloroform-d) d = 7.26 - 7.20 (m, 6 H), 7.16 - 7.13 (m, 1 H), 7.13 - 7.09 (m, 1 H), 6.91 (t, *J* = 7.6 Hz, 1 H), 6.84 (d, *J* = 8.1 Hz, 2 H), 6.78 – 6.73 (m, 2 H), 4.96 (d, *J* = 9.5 Hz, 1 H), 4.80 (s, 1 H), 4.22 (d, *J* = 9.5 Hz, 1 H), 3.83 - 3.75 (m, 4 H), 2.76 (s, 3 H), 1.85 - 1.78 (m, 4 H), 1.00 (t, *J* = 7.3 Hz, 6 H); $^{13}$C NMR (100 MHz, chloroform-d) d = 156.3, 154.0, 153.5, 147.3, 146.3, 145.9, 145.5, 145.3, 144.8, 144.4, 144.3, 143.1, 142.6, 142.3, 142.1, 142.1, 141.7, 140.9, 140.1, 140.1, 136.6, 135.8, 134.7, 131.3, 130.5, 130.1, 129.1, 126.1, 124.2, 123.9, 122.8, 122.5, 120.5, 119.3, 117.9, 115.1, 108.9, 108.8, 82.9, 70.0, 69.0, 49.5, 44.8, 40.1, 34.7, 31.6, 25.3, 22.7, 22.3, 20.1, 14.1, 11.8, 11.3; HRMS (ESI-TOF) m/z calculated for $C_{95}H_{33}N_3S_2$ + H 1280.2189 [M+H]$^+$, measured 1280.1149 [M+H]$^+$.

### Synthesis of PTZ-TCBD-PTZ-$C_{60}$

In a 100 mL round-bottomed flask, compound **PTZ-TCBD-PTZ** (110 mg, 0.1665 mmol), $C_{60}$-fullerene (239 mg, 0.3329 mmol) and *N*-methylglycine (118 mg, 1.332 mmol) were dissolved in toluene (20 mL) and refluxed for 48 h. After that, the reaction mixture was extracted with DCM, washed with brine solution and dried over anhydrous sodium sulfate. The solvent was evaporated under vacuum, and the product was purified by column chromatography with DCM/hexane (1:1, v/v) as the eluent to get the desired compound **PTZ-TCBD-PTZ-$C_{60}$** as a dark color solid with 30 % yield. $^1$H NMR (500 MHz, chloroform-d) d = 7.61 (dd, *J* = 2.4, 8.8 Hz, 1 H), 7.55 (d, *J* = 6.6 Hz, 1 H), 7.32 - 7.19 (m, 4 H), 7.09 (t, *J* = 7.7 Hz, 1 H), 6.97 (d, *J* = 7.6 Hz, 1 H), 6.91 (t, *J* = 7.2 Hz, 1 H), 6.80 (s, 1 H), 6.78 (d, *J* = 4.0 Hz, 1 H), 6.77 - 6.70 (m, 2 H), 4.89 (d, *J* = 9.5 Hz, 1 H), 4.73 (s, 1 H), 4.15 (d, *J* = 9.5 Hz, 1 H), 3.75 (td, *J* = 7.5, 15.3 Hz, 4 H), 2.69 (s, 3 H), 1.81 - 1.74 (m, 4 H), 0.96 (t, *J* = 7.3 Hz, 6 H); $^{13}$C NMR (100 MHz, chloroform-d) d = 165.9, 164.0, 155.1, 152.9, 151.9, 149.4, 146.3, 145.5, 145.3, 145.2, 145.1, 145.0, 144.7, 144.5, 144.3, 144.3, 143.7, 143.5, 143.4, 143.3, 143.0, 142.1, 142.0, 141.7, 141.6, 141.2, 141.1, 141.0, 141.0, 140.8, 140.7, 140.6, 140.4, 139.2, 139.0, 138.6, 136.0, 135.4, 134.9, 134.6, 132.5, 129.8, 126.7, 126.6, 126.5, 124.2, 123.8, 123.2, 122.3, 121.5, 120.9, 120.3, 120.2, 114.8, 113.8, 112.6, 112.1, 111.5, 111.2, 109.2, 108.9, 81.5, 80.0, 79.5, 68.9, 68.0, 49.2, 44.2, 39.0, 28.7, 21.3, 18.9, 13.1, 10.8, 10.1; MALDI calculated for $C_{101}H_{33}N_7S_2$ 1407.2239 [M]$^+$; measured 1407.043.

**Synthesis of PTZ-DCNQ-PTZ-$C_{60}$**

In a 100 mL round-bottomed flask, compound **PTZ-DCNQ-PTZ** (80 mg, 0.1085 mmol), $C_{60}$-fullerene (156 mg, 0.2171 mmol) and N-methylglycine (77 mg, 0.8684 mmol) were dissolved in toluene (20 mL) and refluxed for 60 h. After that, the reaction mixture was extracted with DCM, washed with brine solution and dried over anhydrous sodium sulfate. The solvent was evaporated under vacuum, and the product was purified by column chromatography with DCM/hexane (1:1, v/v) as the eluent to get the desired compound **PTZ-DCNQ-PTZ-$C_{60}$** as a dark color solid with 35 % yield. $^{1}$H NMR (500 MHz, chloroform-d) d = 7.53 (d, *J* = 8.5 Hz, 1 H), 7.48 - 7.44 (m, 1 H), 7.35 (d, *J* = 2.4 Hz, 2 H), 7.31 (br. s., 1 H), 7.28 (d, *J* = 2.0 Hz, 1 H), 7.21 - 7.16 (m, 1 H), 7.16 - 7.11 (m, 2 H), 7.10 - 7.05 (m, 2 H), 6.98 - 6.92 (m, 3 H), 6.86 – 6.80 (m, 2 H), 6.74 (d, *J* = 9.0 Hz, 1 H), 4.95 (d, *J* = 9.5 Hz, 1 H), 4.79 (s, 1 H), 4.21 (d, *J* = 9.5 Hz, 1 H), 3.83 - 3.74 (m, 4 H), 2.77 - 2.73 (m, 3 H), 1.87 - 1.78 (m, 4 H), 1.03 - 0.99 (m, 6 H); $^{13}$C NMR (100 MHz, chloroform-d) d = 167.9, 156.1, 154.1, 153.9, 153.2, 149.5, 149.2, 149.2, 148.7, 147.4, 147.3, 147.1, 146.5, 146.4, 146.3, 146.2, 146.1, 146.0, 145.7, 145.5, 145.5, 145.4, 145.3, 145.3, 145.2, 144.8, 144.5, 144.4, 144.3, 143.2, 143.0, 142.8, 142.7, 142.6, 142.2, 142.2, 142.2, 142.1, 142.0, 141.7, 141.6, 140.2, 140.0, 139.6, 139.0, 137.0, 135.9, 135.7, 134.7, 134.1, 133.2, 133.2, 131.9, 130.7, 129.2, 129.0, 127.9, 127.8, 127.6, 127.0, 126.2, 126.1, 125.6, 124.6, 124.5, 124.4, 124.0, 123.9, 123.5, 122.9, 119.0, 118.9, 116.0, 115.3, 114.8, 113.8, 113.6, 113.1, 82.5, 81.7, 75.7, 69.9, 69.0, 50.2, 49.8, 40.0, 35.0, 34.9, 34.5, 34.4, 33.5, 31.9, 31.5, 31.5, 30.2, 30.2, 29.7, 29.6, 29.5, 29.4, 29.3, 29.1, 29.1, 24.8, 22.7, 20.1, 19.9, 14.1, 11.3, 11.1; MALDI calculated for $C_{107}H_{37}N_7S_2$ 1483.2552 $[M]^+$; measured 1483.123.

**$^{1}$H-NMR, $^{13}$C-NMR and HRMS or MALDI for PTZ-PTZ, PTZ-TCBD-PTZ, PTZ-DCNQ-PTZ, PTZ-PTZ-$C_{60}$, PTZ-TCBD-PTZ-$C_{60}$, and PTZ-DCNQ-PTZ-$C_{60}$**

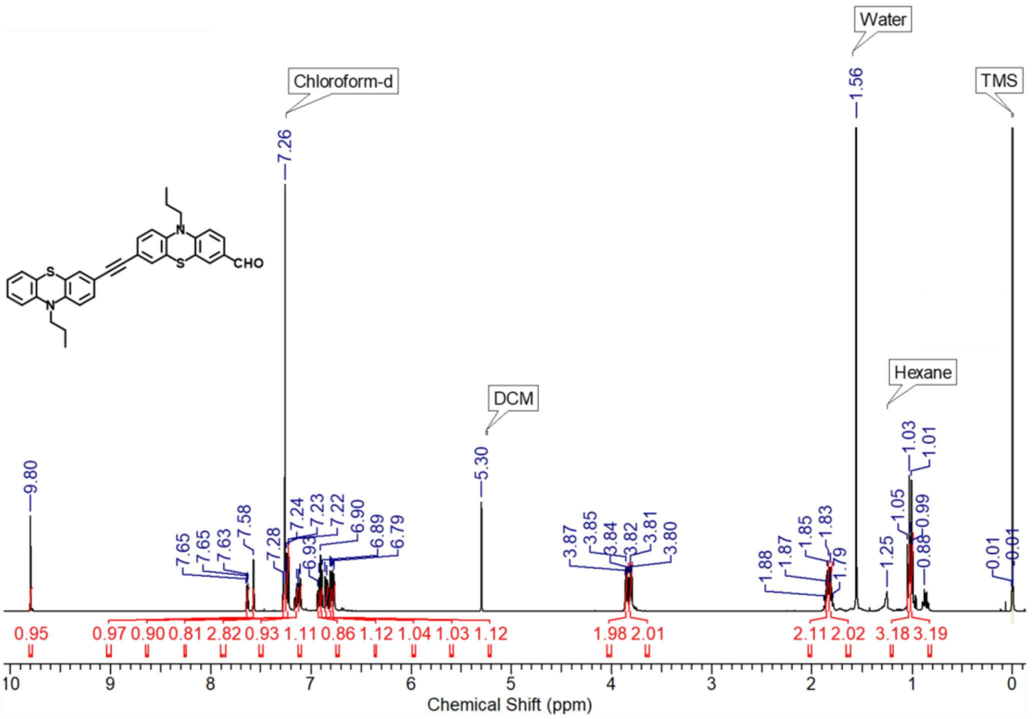


**Figure S1.** $^{1}$H-NMR of **PTZ-PTZ**

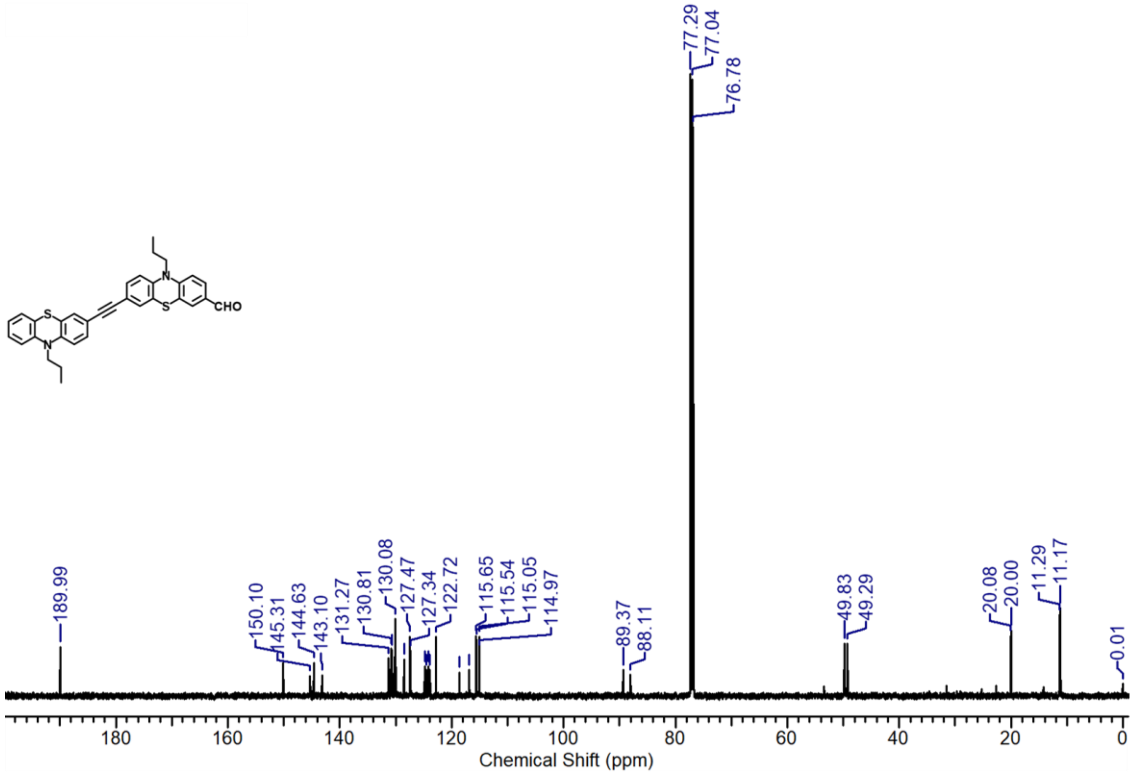


**Figure S2.** $^{13}$C-NMR of **PTZ-PTZ**

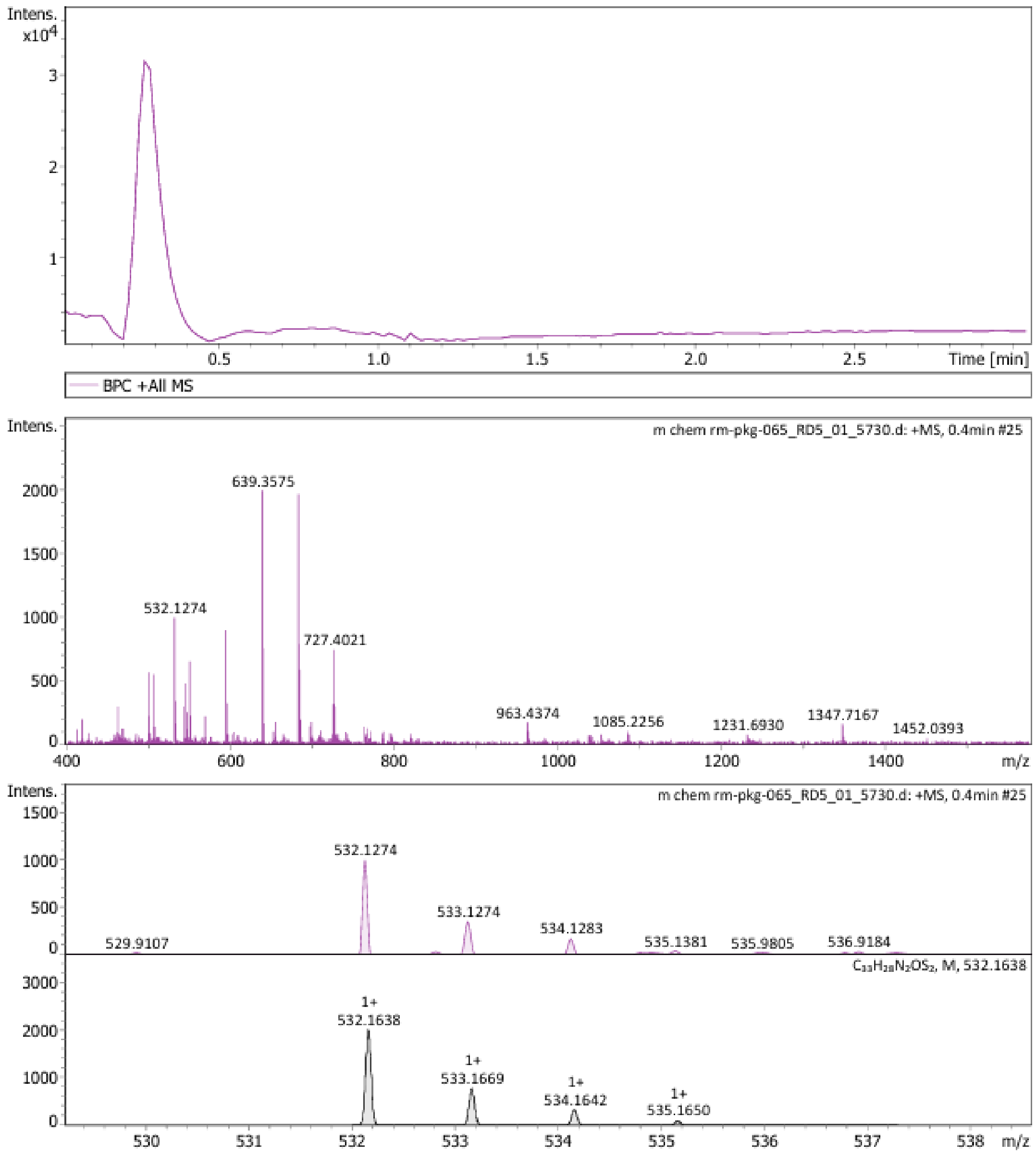


**Figure S3**. HRMS of **PTZ-PTZ**

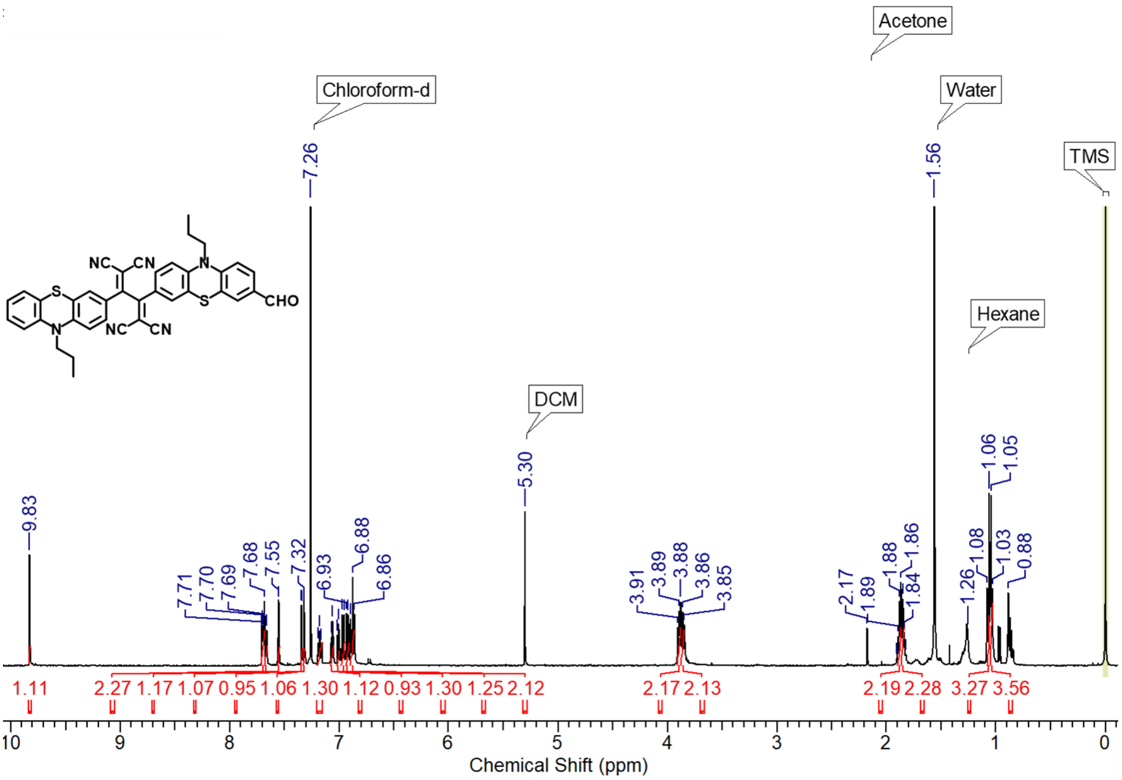


**Figure S4.** $^{1}$H-NMR of **PTZ-TCBD-PTZ**

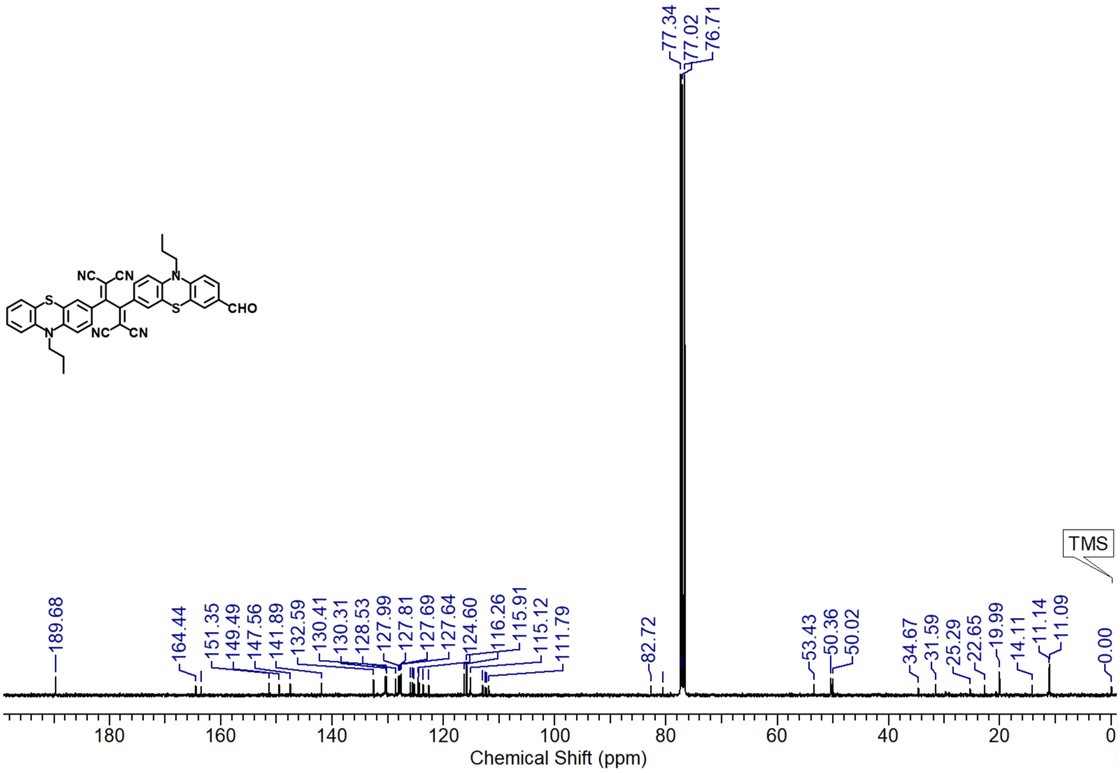


**Figure S5.** $^{13}$C-NMR of **PTZ-TCBD-PTZ**

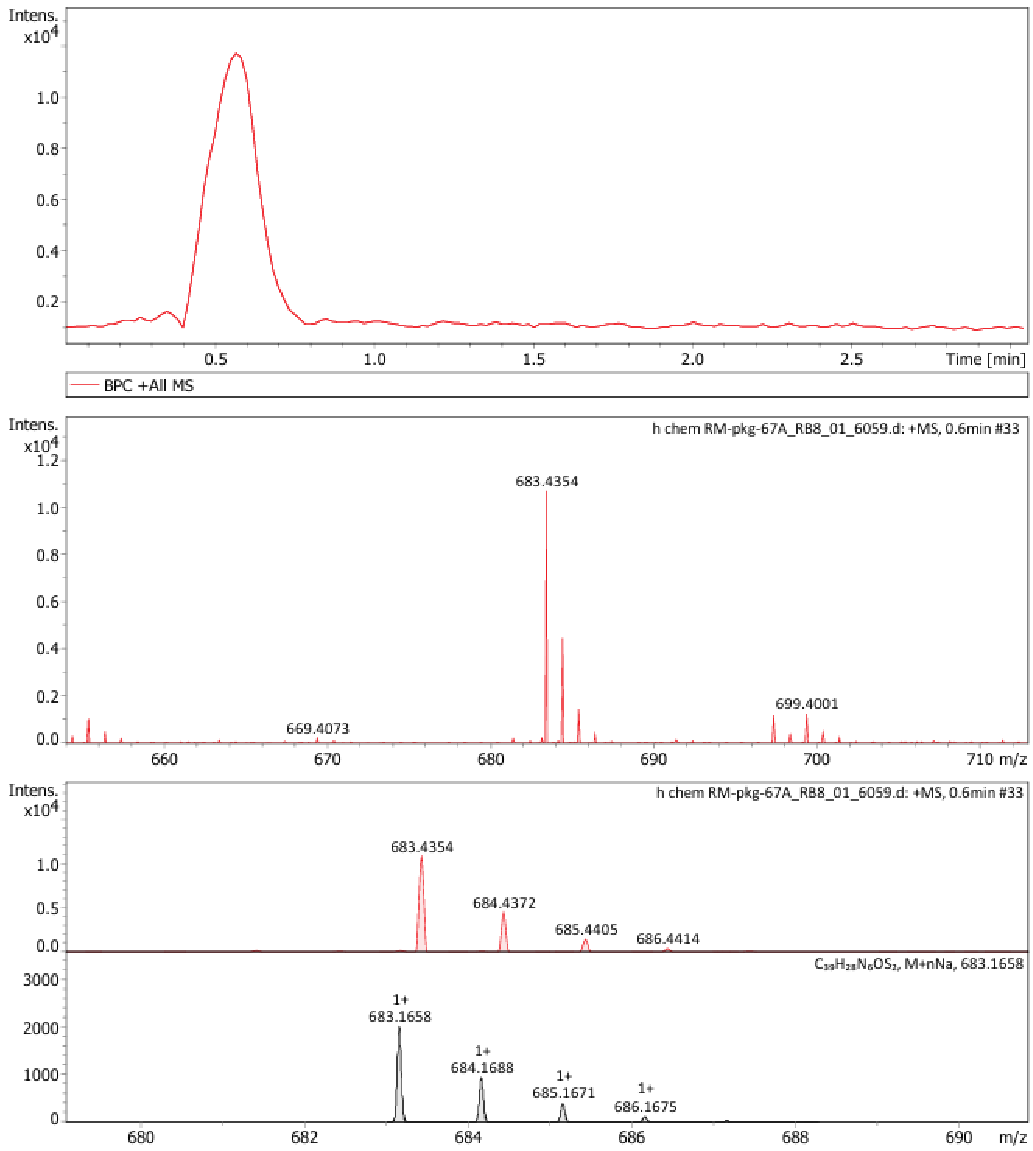


**Figure S6.** HRMS of **PTZ-TCBD-PTZ**

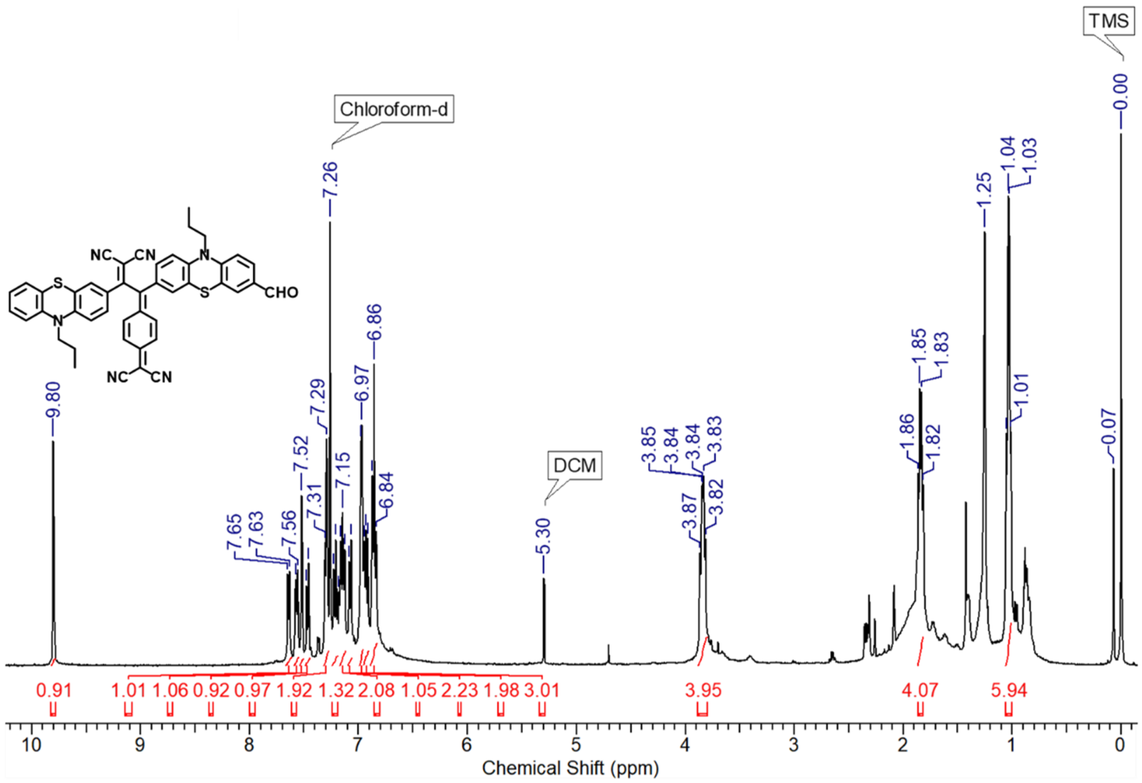

**Figure S7.** $^{1}$H-NMR of **PTZ-DCNQ-PTZ**

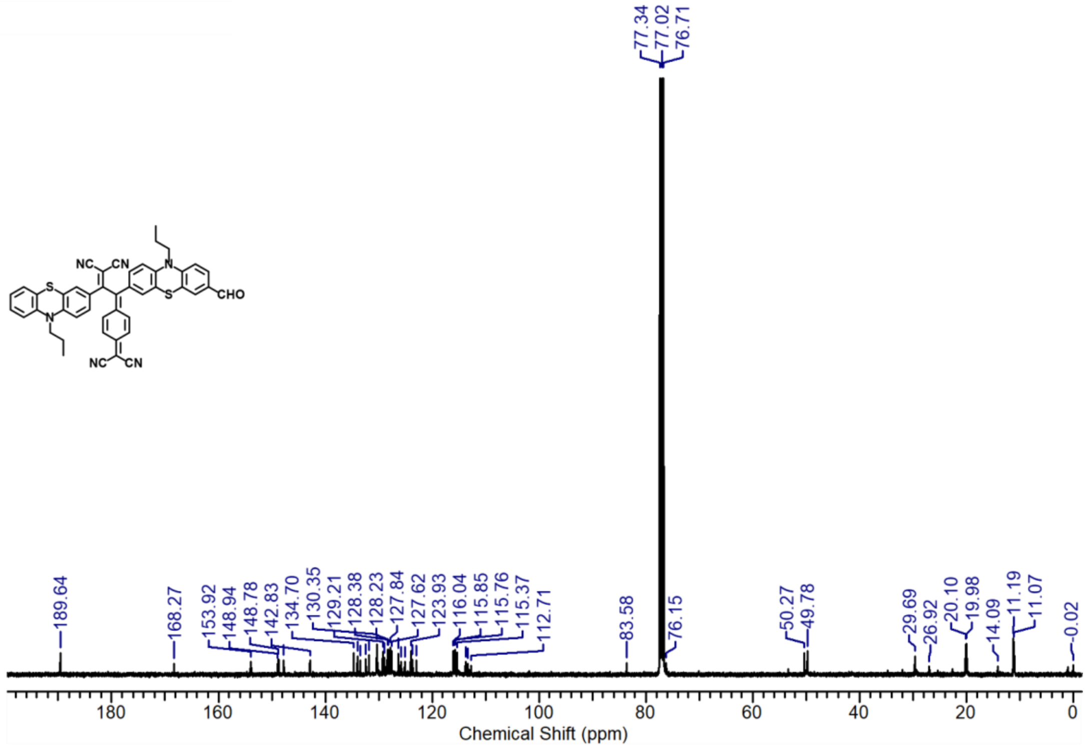

**Figure S8.** $^{13}$C-NMR of **PTZ-DCNQ-PTZ**

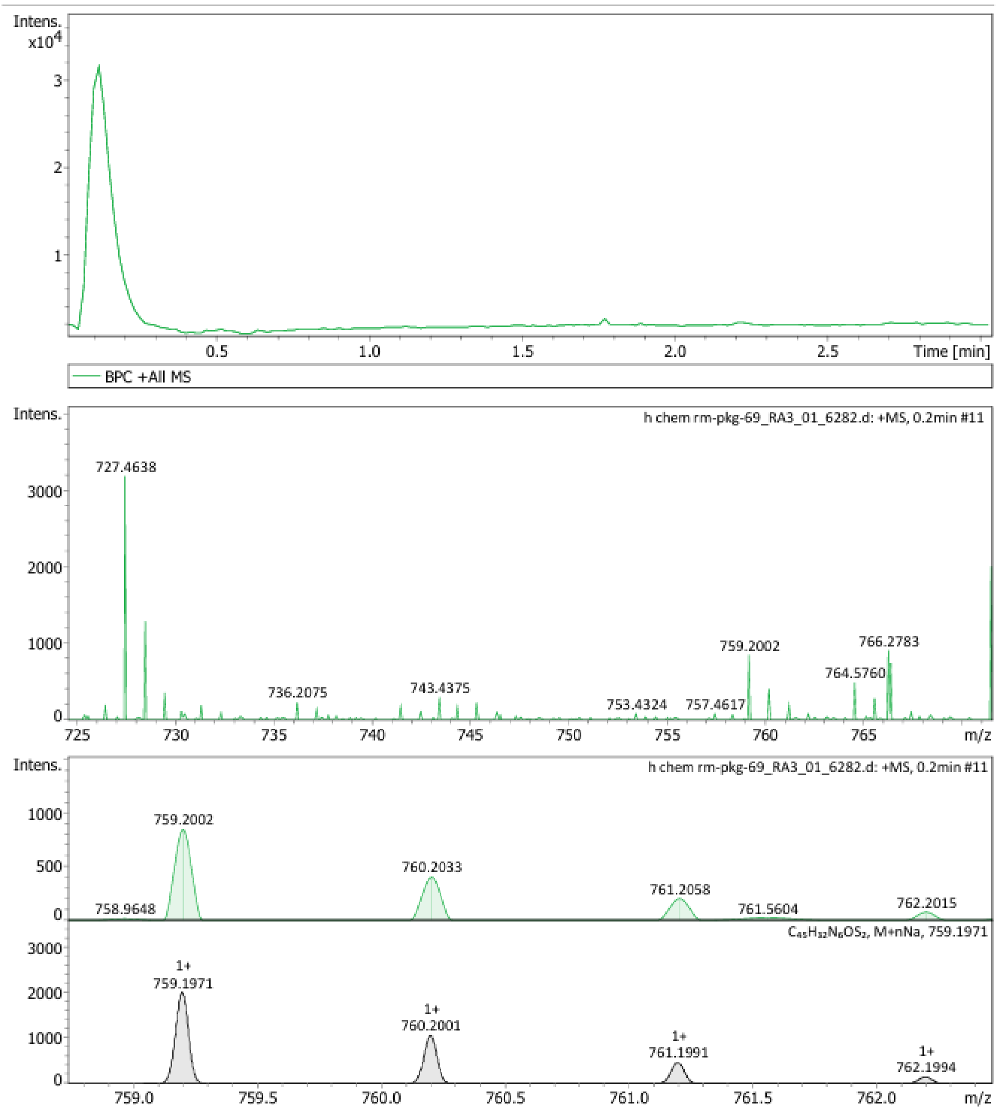


**Figure S9.** HRMS of **PTZ-DCNQ-PTZ**

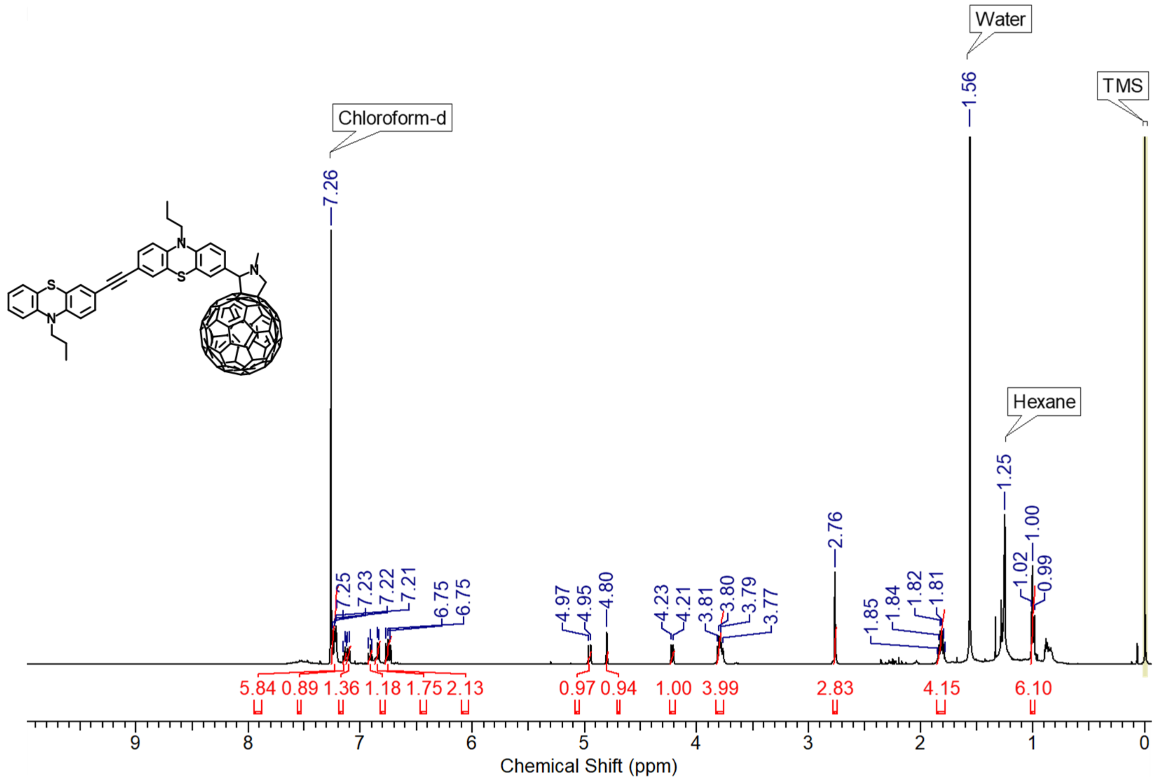


**Figure S10.** $^{1}$H-NMR of **PTZ-PTZ-C$_{60}$**

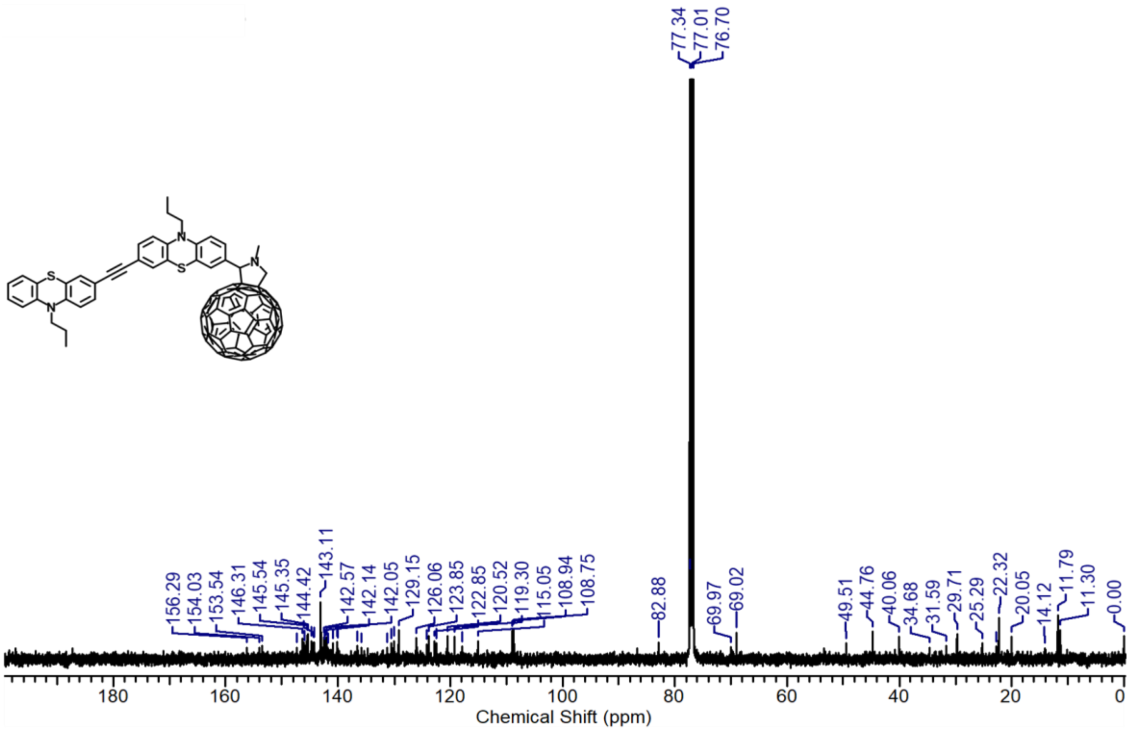


**Figure S11.** $^{13}$C-NMR of **PTZ-PTZ-C$_{60}$**

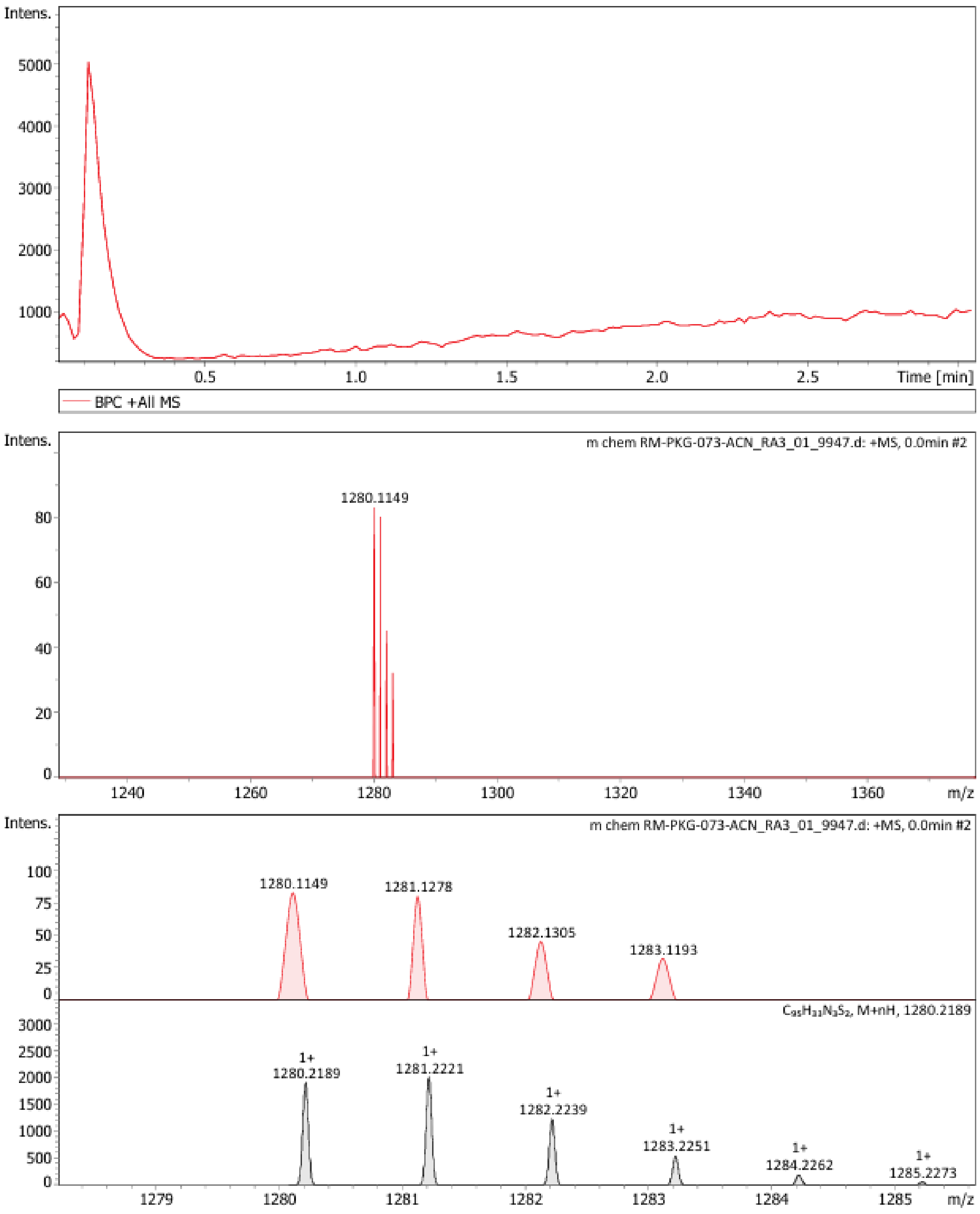


**Figure S12.** HRMS of **PTZ-PTZ-C$_{60}$**

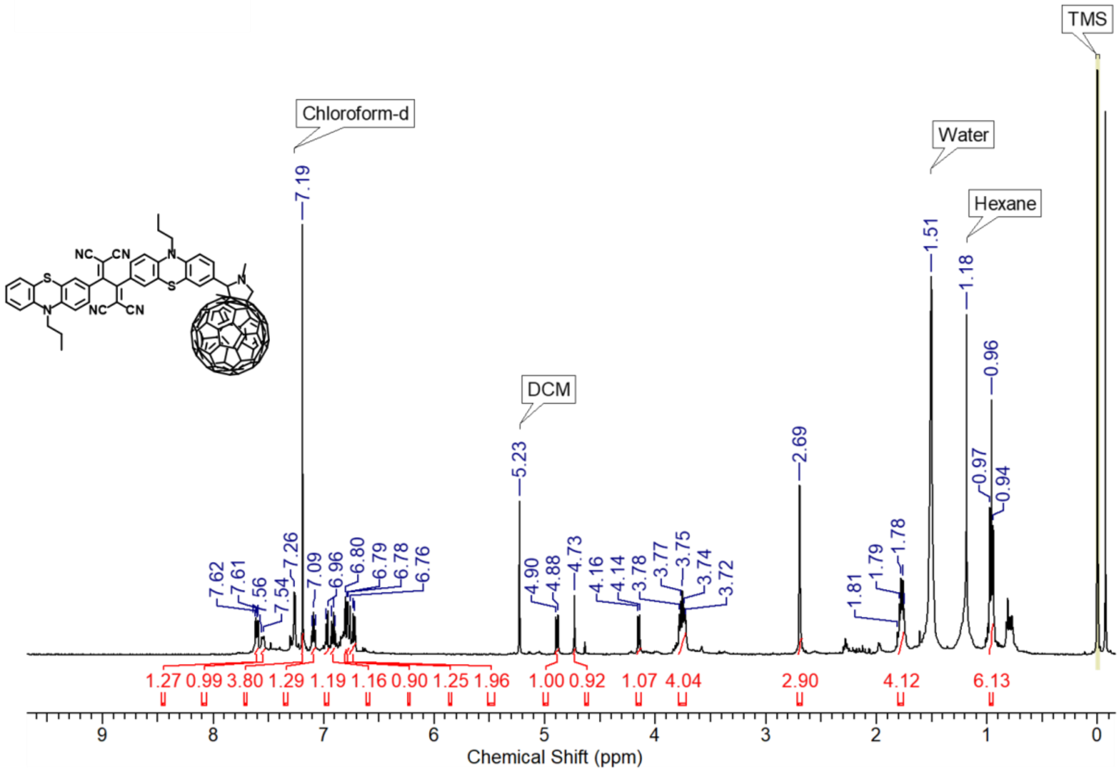


**Figure S13.** $^{1}$H-NMR of **PTZ-TCBD-PTZ-$C_{60}$**

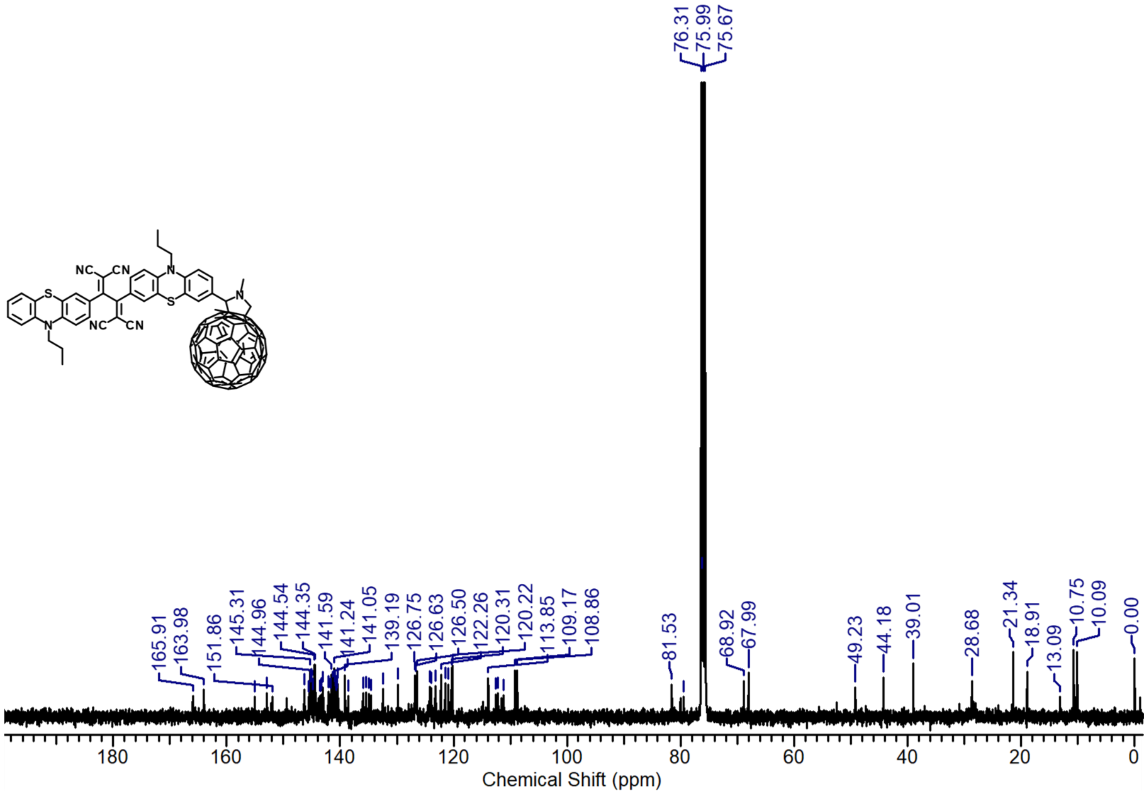


**Figure S14.** $^{13}$C-NMR of **PTZ-TCBD-PTZ-$C_{60}$**

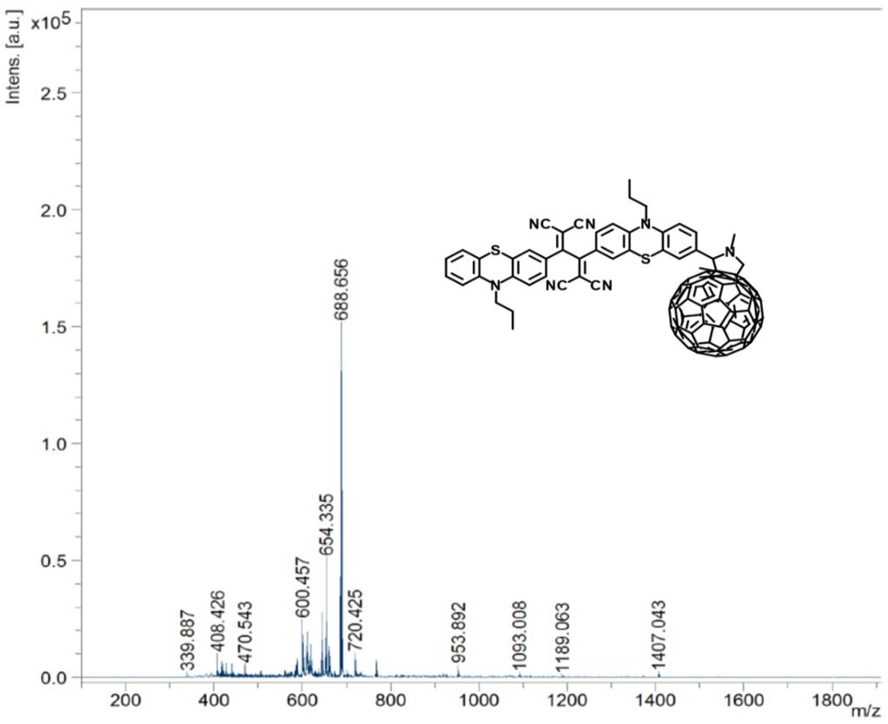


**Figure S15.** MALDI of **PTZ-TCBD-PTZ-$C_{60}$**

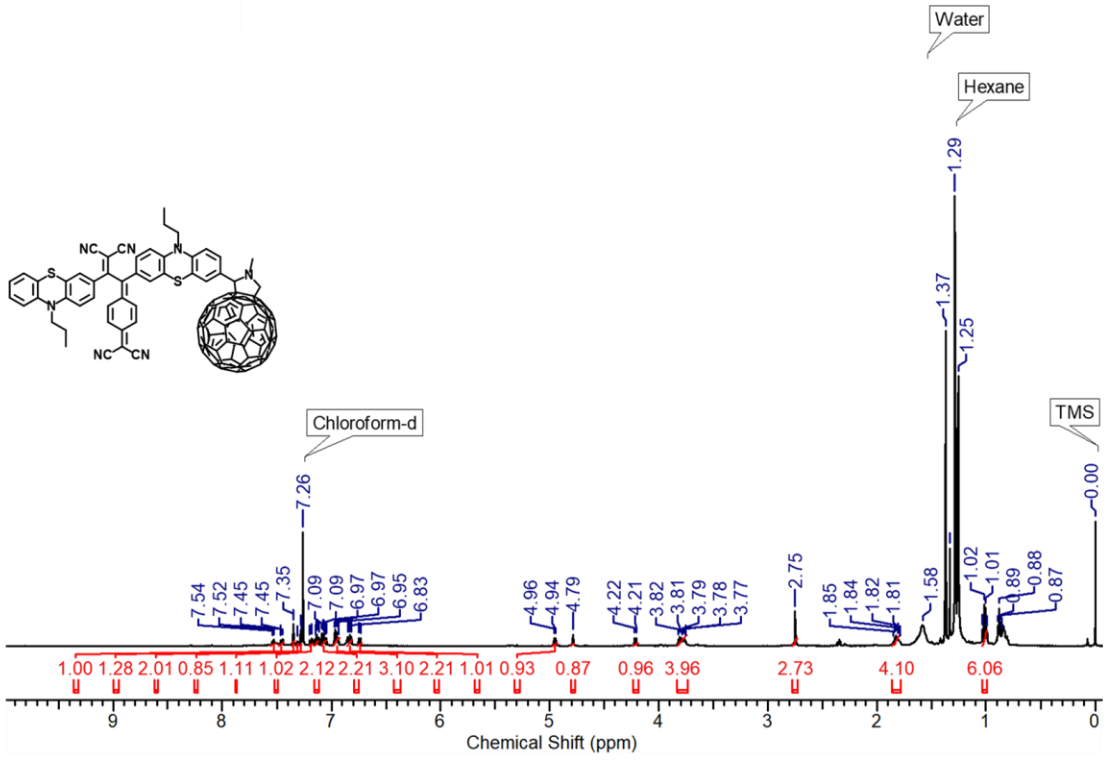


**Figure S16.** $^{1}$H-NMR of **PTZ-DCNQ-PTZ-$C_{60}$**

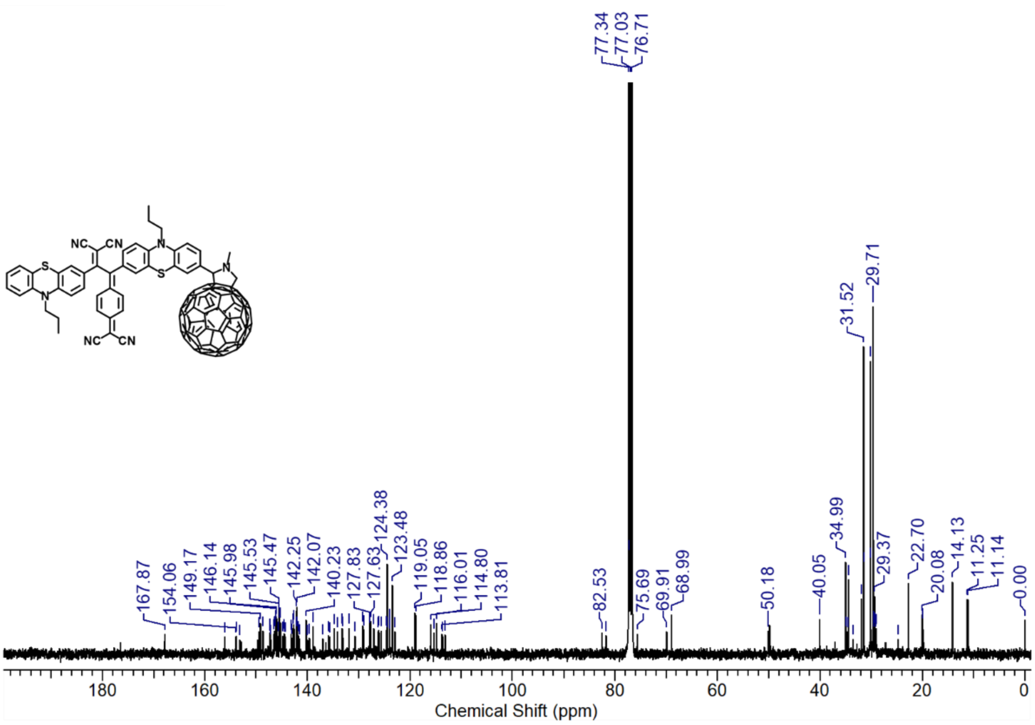


**Figure S17.** $^{13}$C-NMR of **PTZ-DCNQ-PTZ-$C_{60}$**

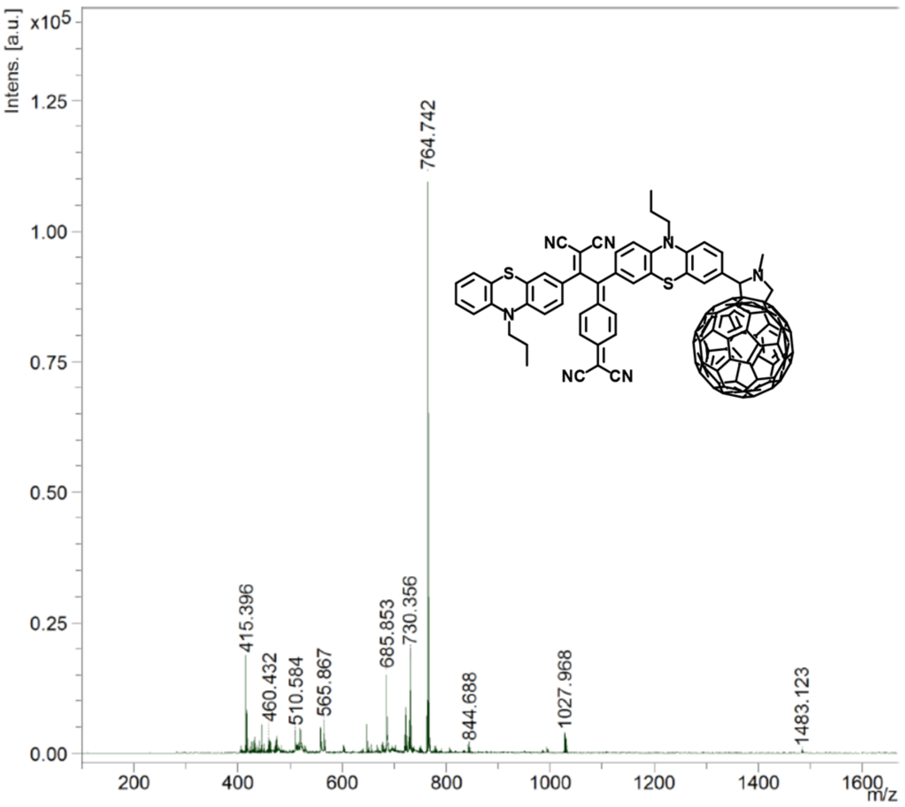


**Figure S18.** MALDI of **PTZ-DCNQ-PTZ-$C_{60}$**

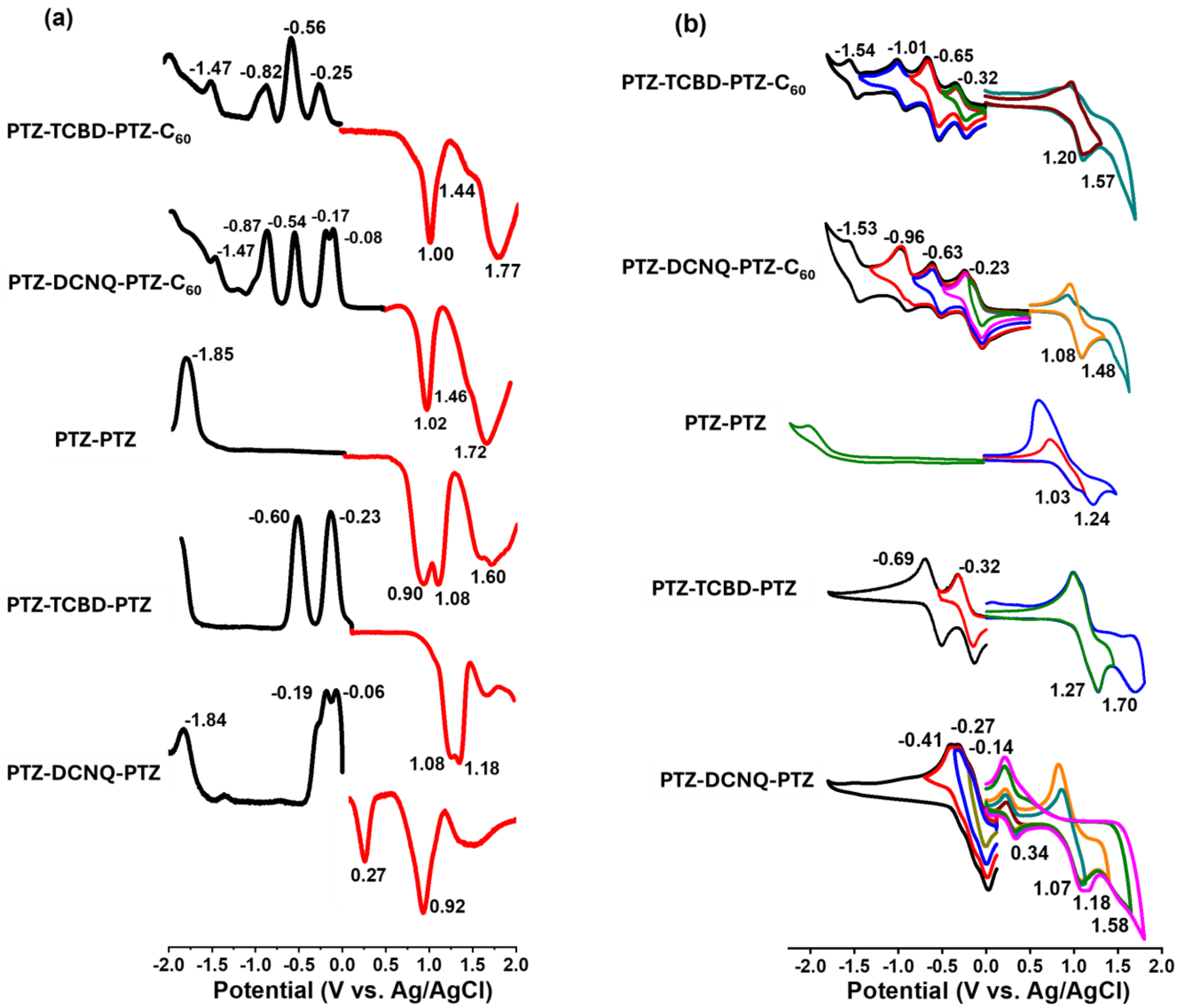


**Figure S19.** (a) Differential pulse voltammograms (DPV) and (b) cyclic voltammograms (CV) of compounds **PTZ-TCBD-PTZ-$C_{60}$, PTZ-DCNQ-PTZ-$C_{60}$, PTZ-PTZ , PTZ-TCBD-PTZ,** and **PTZ-DCNQ-PTZ** in dichlorobenzene with 0.1 M (TBA)$ClO_4$ (tetrabutylammonium perchlorate) by using three electrode system (Ag/AgCl as reference electrode, Pt as a working, and counter electrodes).

Table S1. Redox potentials, free-energy change for charge transfer ($\Delta G_{CT}$), separation ($\Delta G_{CS}$) and recombination ($\Delta G_{CR}$) for the investigated compounds in 1,2-dichlorobenzene.

| Compound | Reduction potential | | | Oxidation potential | | -$\Delta G_{CT}$, eV | -$\Delta G_{CS}$, eV | -$\Delta G_{CR}$, eV |
|---|---|---|---|---|---|---|---|---|
| | 3rd red. | 2nd red. | 1st red. | 1st oxid. | 2nd oxid. | | | |
| PTZ-PTZ-$C_{60}$ | -1.33 | -0.98 | -0.60 | 0.85 | 0.96 | 1.45 | 1.40 | 1.35 |
| PTZ-TCBD-PTZ-$C_{60}$ | -0.82 | -0.56 | -0.25 | 1.00 | 1.44 | 1.25 | 1.72 | 1.15 |
| PTZ-DCNQ-PTZ-$C_{60}$ | -0.54 | -0.17 | -0.08 | 1.02 | 1.46 | 1.10 | 1.29 | 0.91 |
| PTZ-PTZ | - | - | -1.85 | 0.90 | 1.08 | - | - | - |
| PTZ-TCBD-PTZ | - | -0.60 | -0.23 | 1.08 | 1.18 | - | - | - |
| PTZ-DCNQ-PTZ | -1.84 | -0.19 | -0.06 | 0.27 | 0.92 | - | - | - |

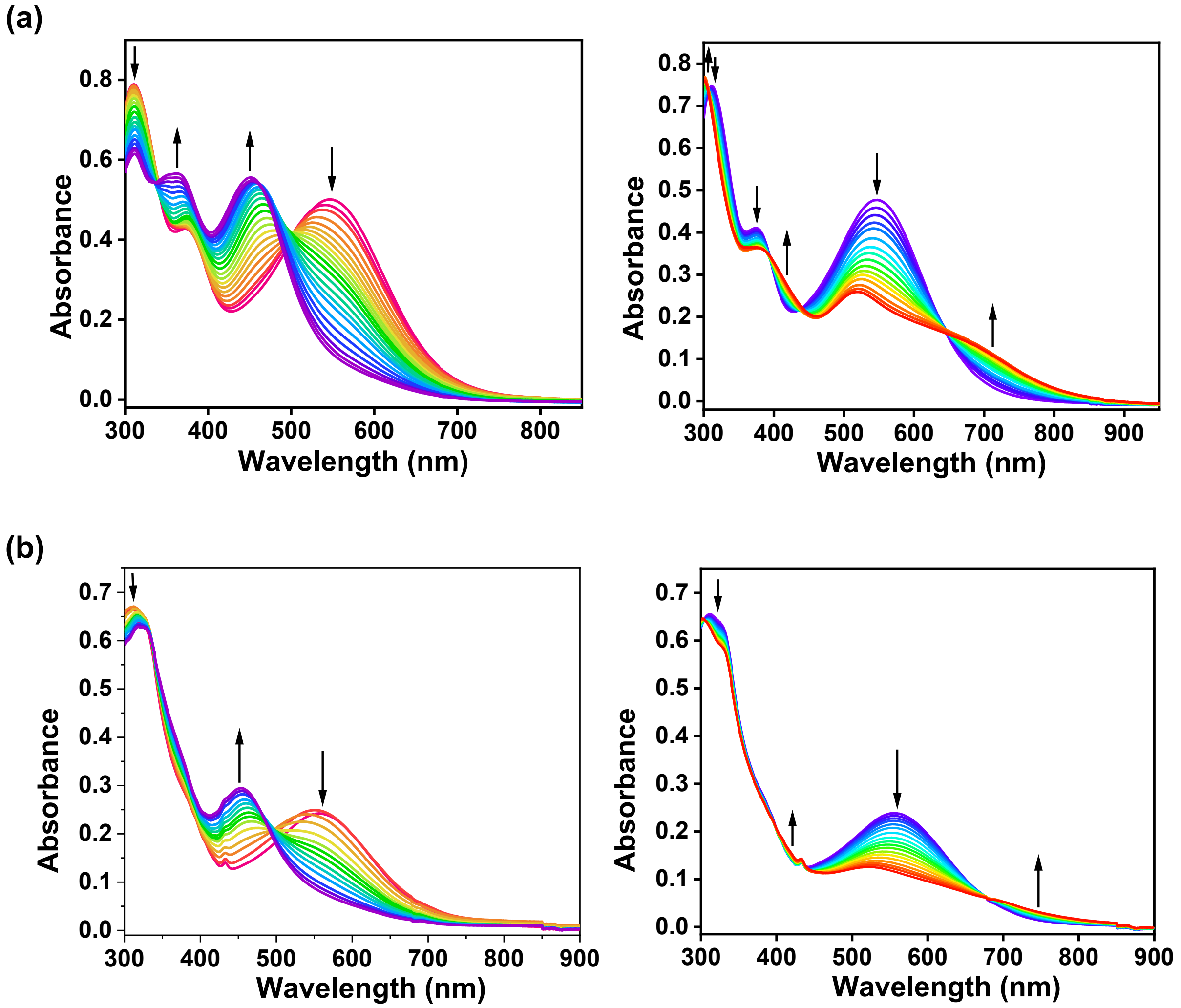


**Figure S20.** Spectral changes observed during left: first oxidation and right: first reduction of (a) **PTZ-TCBD-PTZ** and (b) **PTZ-TCBD-PTZ-$C_{60}$** in dichlorobenzene with 0.2 M (TBA)$ClO_4$. Spectra were recorded until no additional spectral changes were observed.

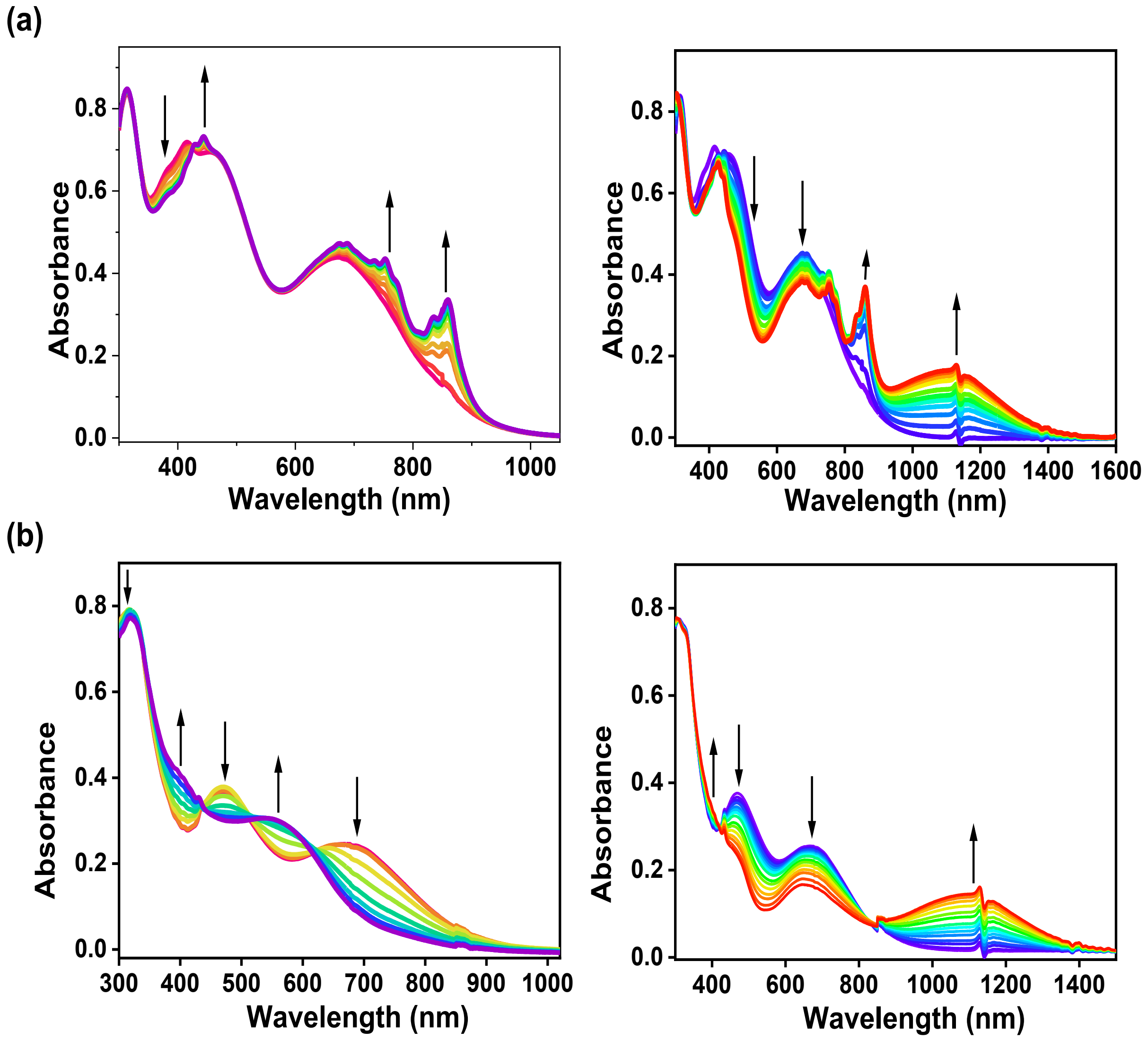


**Figure S21.** Spectral changes observed during left: first oxidation and right: first reduction of (a) **PTZ-DCNQ-PTZ** and (b) **PTZ-DCNQ-PTZ-$C_{60}$** in dichlorobenzene with 0.2 M (TBA)$ClO_4$. Spectra were recorded until no additional spectral changes were observed.

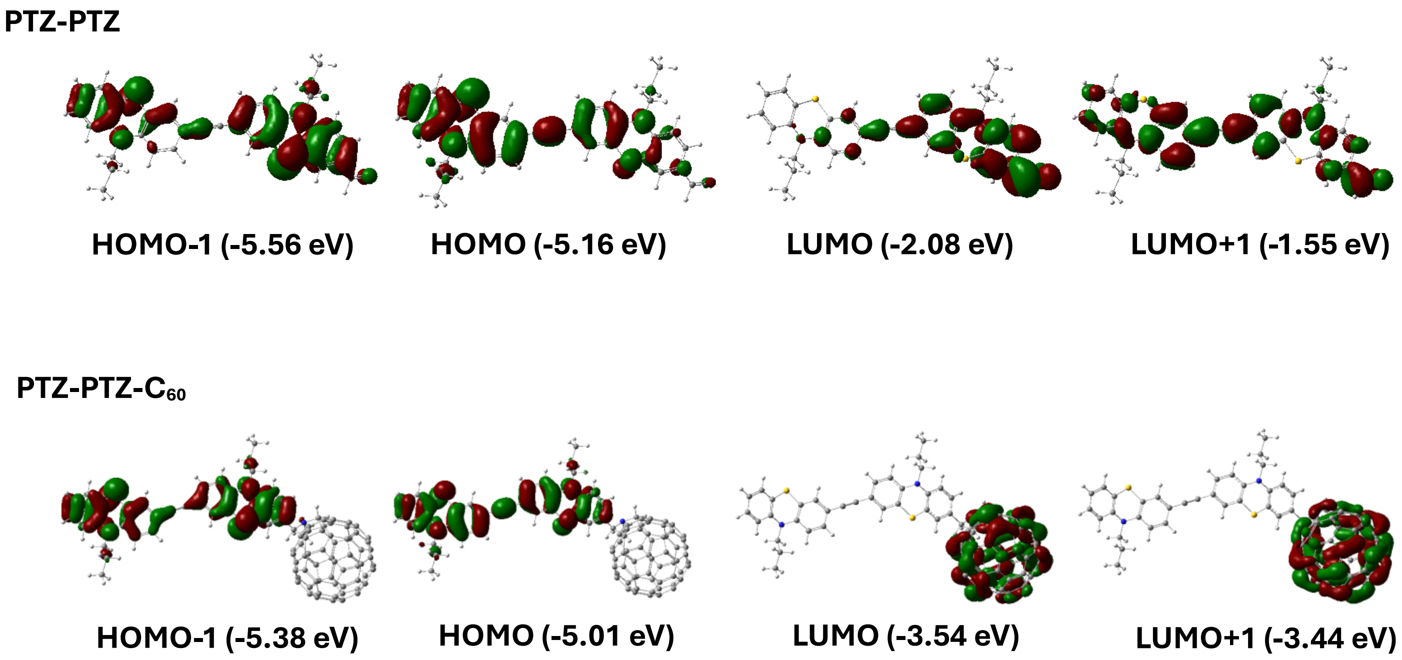


**Figure S22.** Frontier HOMOs and LUMOs and their energies of the compound **PTZ-PTZ** and **PTZ-PTZ-$C_{60}$** generated on DFT/B3LYP/6-311+G (d, p) optimized geometries in gas phase.

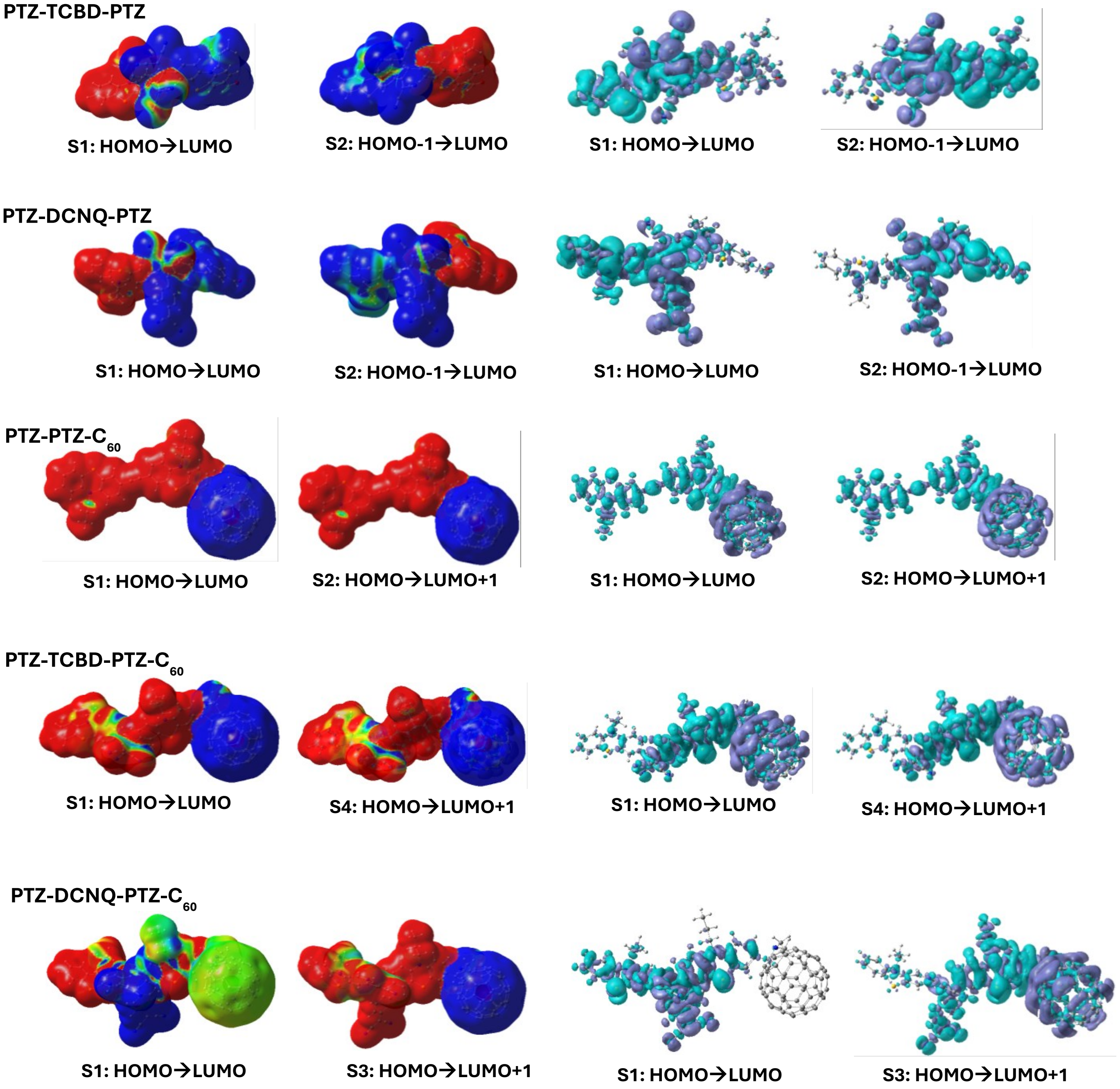


**Figure S23.** Electrostatic Potential Surfaces (Red = electron-rich regions, Blue = electron-poor regions (scale -5.000 x $10^{-2}$ V – 5.000 x $10^{2}$ V)) and the charge transfer locations for the investigated compounds on B3LYP/6-311+G(d,p) optimized structures in the gas phase.

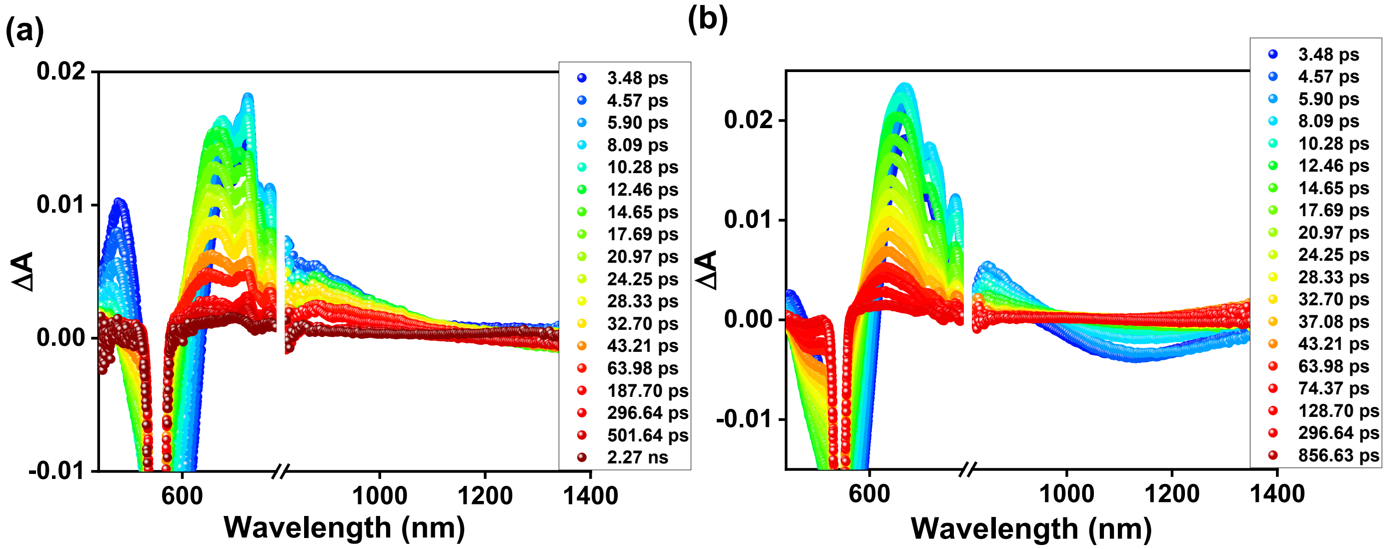


**Figure S24**. Fs-TA Spectra at indicated delay times of (a) **PTZ-TCBD-PTZ-$C_{60}$** and (b) **PTZ-TCBD-PTZ** in 1,2-dichlorobenzene ($\lambda_{ex}$ = 555 nm).

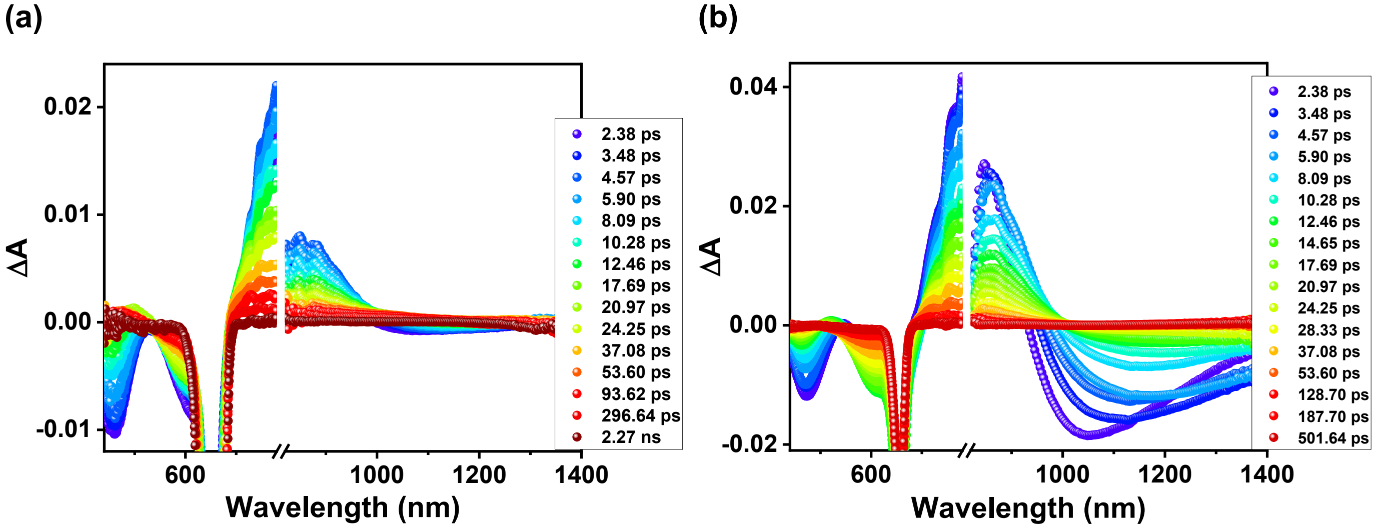


**Figure S25.** Fs-TA Spectra at indicated delay times of (a) **PTZ-DCNQ-PTZ-$C_{60}$** and (b) **PTZ-DCNQ-PTZ** in 1,2-dichlorobenzene ($\lambda_{ex}$ = 665 nm).

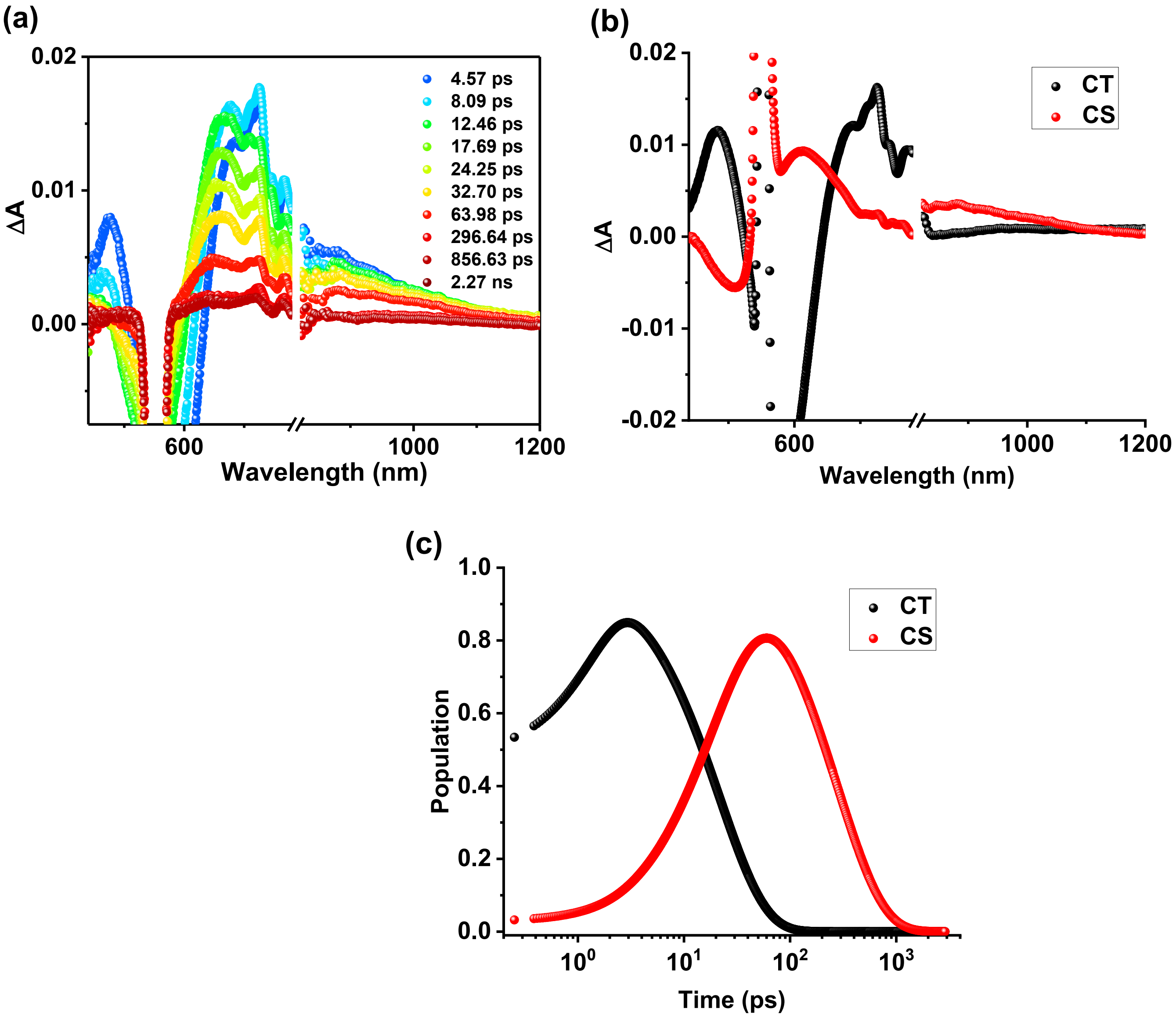


**Figure S26.** (a) Fs-TA Spectra at indicated delay times, (b) decay-associated spectra (DAS), and (c) population kinetics from GloTarAn analysis of **PTZ-TCBD-PTZ-$C_{60}$** in 1,2-dichlorobenzene ($\lambda_{ex}$ =555 nm).

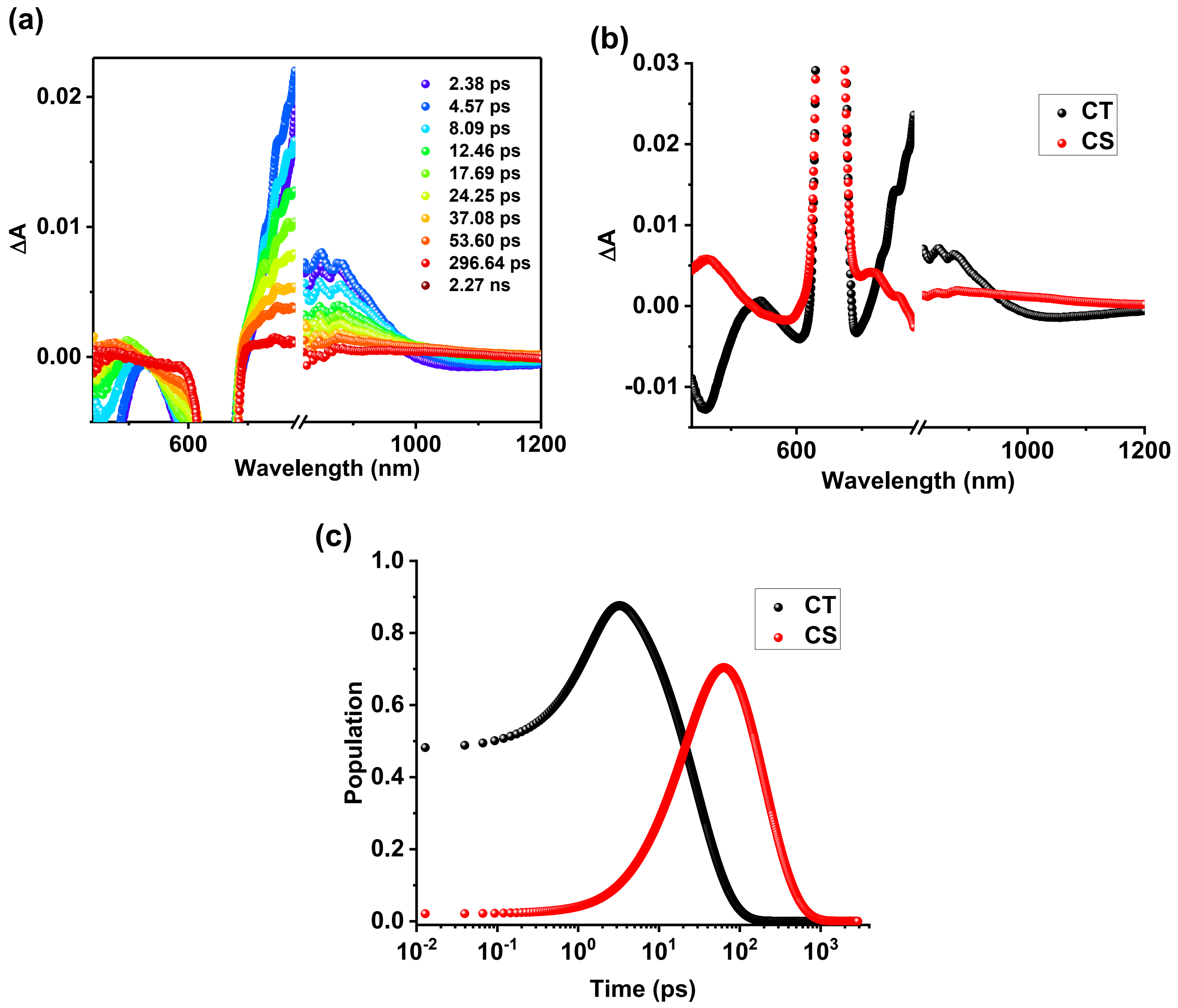


**Figure S27.** (a) Fs-TA Spectra at indicated delay times, (b) decay-associated spectra (DAS), and (c) population kinetics from GloTarAn analysis of **PTZ-DCNQ-PTZ-$C_{60}$** in 1,2-dichlorobenzene ($\lambda_{ex}$ =665 nm).

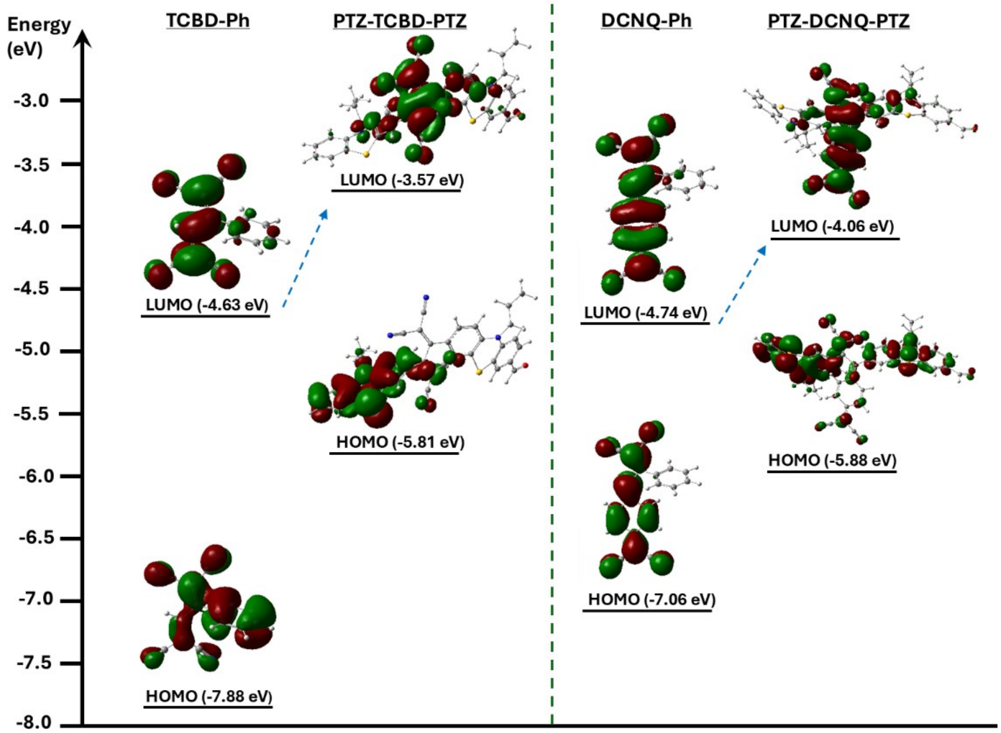


**Figure S28**. Comparison of HOMO and LUMO orbital distribution and energies of TCBD-Ph and PTZ-TCBD-PTZ, and DCNQ-Ph and PTZ-DCNQ-PTZ. The higher energy of LUMO in PTZ-TCBD-PTZ and PTZ-DCNQ-PTZ suggests it is a poor electron acceptor.

## References


1. R. Sharma, M. B. Thomas, R. Misra and F. D'Souza, *Angew. Chem. Int. Ed.*, 2019, **58**, 4350–4355.
2. D. Pinjari, A. Z. Alsaleh, Y. Patil, R. Misra and F. D'Souza, *Angew. Chem. Int. Ed.*, 2020, **59**, 23697–23705.
3. I. S. Yadav, A. Z. Alsaleh, R. Misra and F. D'Souza, *Chem. Sci.*, 2021, **12**, 1109–1120.
